# Revisiting Dipole-Induced Fluorinated-Anion Decomposition Reaction for Promoting a LiF-Rich Interphase in Lithium-Metal Batteries

**DOI:** 10.1007/s40820-024-01637-5

**Published:** 2025-01-20

**Authors:** Liu Wang, Jiahui Guo, Qi Qi, Xiaotong Li, Yuanmeng Ge, Haoyi Li, Yunfeng Chao, Jiang Du, Xinwei Cui

**Affiliations:** 1 Henan Institutes of Advanced Technology, Zhengzhou University, Zhengzhou, 450003 People’s Republic of China; 2 College of Materials Science and Engineering, Zhengzhou University, Zhengzhou, 450001 People’s Republic of China; 3 State Key Laboratory of Coking Coal Resources Green Exploitation, Zhengzhou University, Zhengzhou, 450001 People’s Republic of China

**Keywords:** LiF-rich SEI, Li-ion mobility, Lithiophilicity, MXene, Graphdiyne

## Abstract

Building anion-derived solid electrolyte interphase (SEI) with enriched LiF is considered the most promising strategy to address inferior safety features and poor cyclability of lithium-metal batteries (LMBs). Herein, we discover that, instead of direct electron transfer from surface polar groups to bis(trifluoromethanesulfonyl)imide (TFSI^−^) for inducing a LiF-rich SEI, the dipole-induced fluorinated-anion decomposition reaction begins with the adsorption of Li ions and is highly dependent on their mobility on the polar surface. To demonstrate this, a single-layer graphdiyne on MXene (sGDY@MXene) heterostructure has been successfully fabricated and integrated into polypropylene separators. It is found that the adsorbed Li ions connect electron-donating sGDY@MXene to TFSI^−^, facilitating interfacial charge transfer for TFSI^−^ decomposition. However, this does not capture the entire picture. The sGDY@MXene also renders the adsorbed Li ions with high mobility, enabling them to reach optimal reaction sites and expedite their coordination processes with O on O=S=O and F on the broken –CF_3_^−^, facilitating bond cleavage. In contrast, immobilized Li ions on the more lithiophilic pristine MXene retard these cleavage processes. Consequently, the decomposition reaction is accelerated on sGDY@MXene. This work highlights the dedicate balance between lithiophilicity and Li-ion mobility in effectively promoting a LiF-rich SEI for the long-term stability of LMBs.

**Supplementary Information:**

The online version contains supplementary material available at 10.1007/s40820-024-01637-5.

## Highlights

- Single-layer graphdiyne on MXene (sGDY@MXene) heterostructure was fabricated and integrated into polypropylene separators, directing a LiF-rich solid electrolyte interphase and long-term stability of lithium-metal anode.
- Instead of direct electron transfer from surface polar groups to fluorinated anions, the adsorbed Li ions on sGDY@MXene act as dynamic bridges collaboratively connecting the electron-donating heterostructure to the anion and its derivatives, facilitating interface charge transfer.
- Dedicate balance between lithiophilicity and high Li-ion mobility is the key to promote the dipole-induced fluorinated-anion decomposition.

**Supplementary Information:**

The online version contains supplementary material available at 10.1007/s40820-024-01637-5.

## Graphical abstract

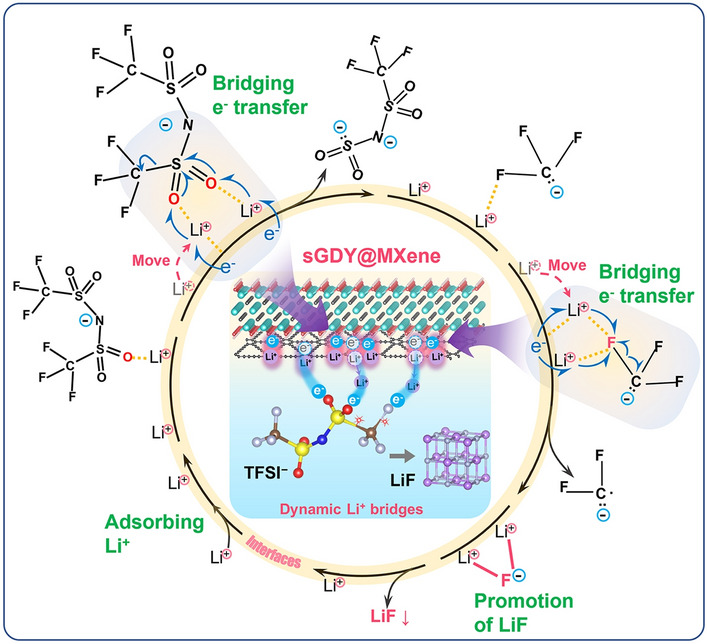

## Introduction

Metallic lithium has long been considered as the “holy grail” of anodes for lithium-based batteries owing to its exceptionally high theoretical capacity of 3860 mAh g^−1^ as well as the most negative redox potential of − 3.04 V versus SHE [1, 2]. However, Li metal has high reactivity with organic electrolytes, spontaneously forming solid electrolyte interphase (SEI) that is derived mainly from the reduction of solvent molecules [3–5]. This solvent-derived SEI has sluggish Li-ion transport and is too fragile to accommodate the rapid growth of Li. Consequently, it becomes unstable during cycling, leading to repeated fracturing/reconstruction and the formation of notorious Li dendrites [6]. As a result, lithium-metal anodes suffer from severe capacity decay and formidable safety hazards, which hinder the practical implementation of lithium-metal batteries (LMBs) [7–9]. Among the efforts to inhibit the formation of Li dendrites, construction of the LiF-rich SEI might be the most promising strategy [10, 11]. Previous studies have shown that LiF-rich SEI possesses several advantageous properties, including high mechanical strength, excellent chemical stability, low solubility, a wide band gap that suppresses electron tunneling, high interfacial energy, and a low Li-ion diffusion barrier [12, 13]. These characteristics effectively mitigate SEI degradation caused by volume expansion, suppress continuous electrolyte decomposition, and promote uniform Li-ion flux within the SEI [14, 15]. Consequently, it facilitates uniform lithium deposition and significantly improves the cycle life of LMBs [16]. LiF in SEI is generally derived from the decomposition of functional fluorinated electrolyte constituents, such as bis(trifluoromethanesulfonyl)imide (TFSI^−^) and bis(fluorosulfonyl)imide (FSI^−^) anions [17, 18]. Therefore, fully understanding the fluorinated-anion decomposition reaction and regulating its reaction kinetics for the formation of LiF-rich SEI is highly desirable, despite being challenging.

Recently, self-assembled polar groups, such as the carboxyl group, have been grafted onto aluminum oxide (Al_2_O_3_)-coated polypropylene (PP) separators [19]. This functional layer has demonstrated enhanced interfacial charge transfer, catalyzing the decomposition of fluorinated anions and inducing the formation of LiF-rich SEI. Following this line of research, the introduction of extended π-conjugated [20] or porphyrin covalent organic framework surfaces [21] has also been demonstrated to donate electrons to the electrolyte environment, improving the kinetics of C–F dissociation. All these strategies involve the establishment of electron-rich surfaces, which conduct electron transfer directly from the surface polar groups to the fluorinated anions through dipole–dipole interactions [22–24]. According to this mechanism, weak dipoles on the surface may fail to facilitate charge transfer across the interface; therefore, it seems that increasingly stronger dipole moments are required for the effective formation of LiF-rich SEI.

However, exceptions also exist. Take MXene nanosheets as an example. MXene nanosheets have been found to possess strong dipole moments on the surface, leading to abundant lithiophilic sites and a high tendency to absorb Li ions [25–27]. This characteristic can guide uniform Li deposition and suppress Li dendrite formation to some extent [28–30]. Nevertheless, a LiF-rich SEI has not been reported for MXene. This discrepancy prompts us to question whether the previous mechanism describes the full picture of the dipole-induced fluorinated-anion decomposition reaction. Since the inevitable attraction of Li ions on the strongly and negatively polarized surfaces, the adsorbed Li ions are expected to play a crucial role for the decomposition of fluorinated anions. Thus, regulation of the adsorbed Li ions on the polar surfaces is necessary to expedite the decomposition reaction. Bearing this objective in mind, the unique structure of graphdiyne (GDY) has captured our attention. GDY is featured with acetylene bonds and highly conjugated pores [31, 32]. These pores can readily anchor heteroatoms or ions, facilitating easy transport of small molecules or ions on the surface [33]. However, GDY is a semiconductive material with relatively weak surface polarization [34]. To take advantages of both MXene and GDY, integrating GDY onto MXene in the formation of a heterostructure could be a viable and effective strategy in modulating the properties of the adsorbed Li ions on the polar surface. Moreover, it is essential to keep the GDY layer as thin as possible to maximize the beneficial effects resulting from the interaction between the two components [35, 36]. Therefore, a single-layer GDY on MXene (sGDY@MXene) heterostructure has been selected as a model material to reveal the reaction landscape of the dipole-induced fluorinated-anion decomposition.

**Fig. 1:**
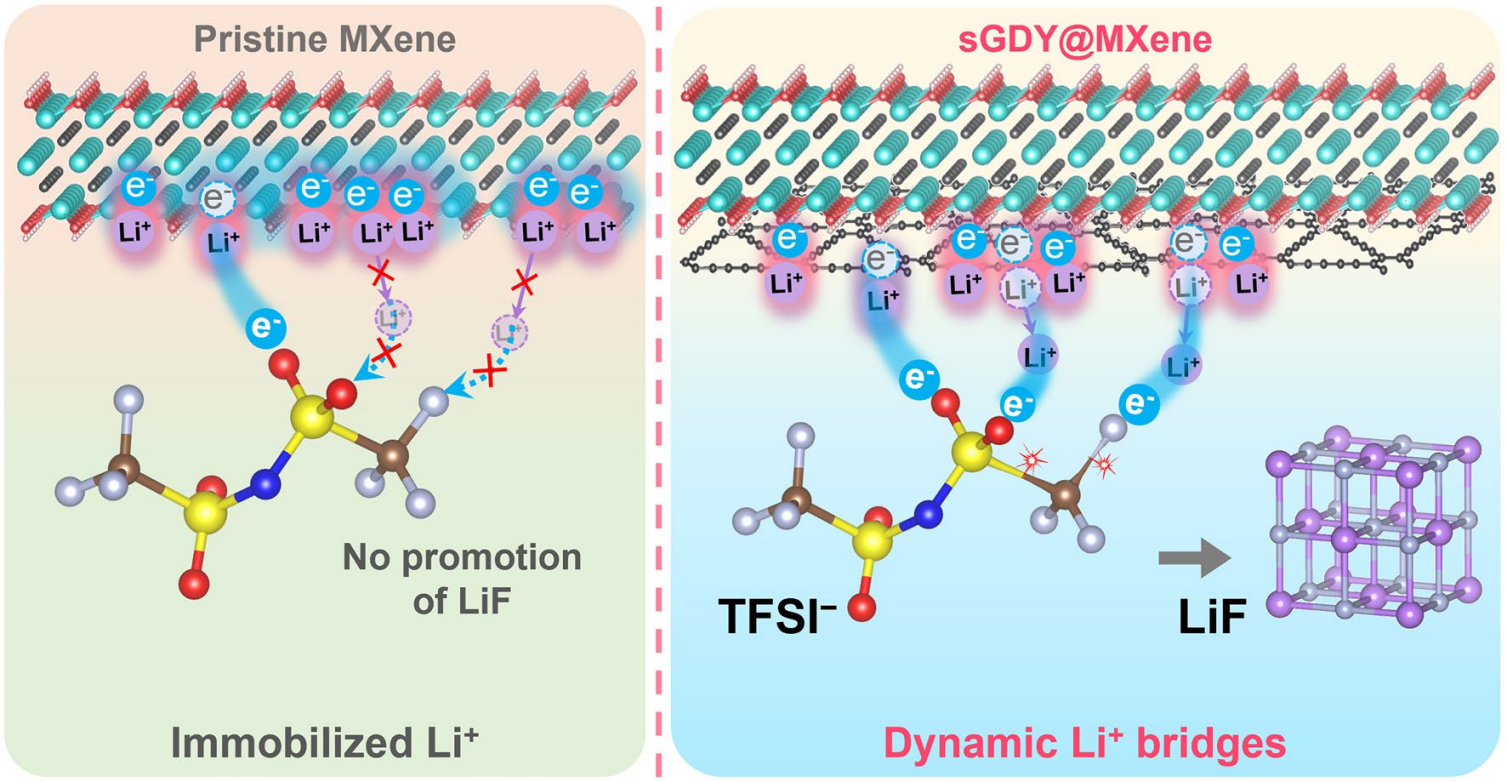
Schematic illustration of the Li-ion-bridged charge transfer process with Li-ion dynamic bridges regulated by sGDY@MXene to promote LiF-rich SEI formation

Herein, sGDY@MXene heterostructured materials have been successfully fabricated through the in situ synthesis of single-layered GDY on Cu^2+^-adsorbed Ti_3_C_2_-MXene nanosheets. Based on this model material, when integrated onto the PP separator as a functional layer, it has been found that, in addition to the sufficient adsorption strength to attract Li ions for bridging the interfacial charge transfer from the electron-donating sGDY@MXene to TFSI^−^, the mobility of the adsorbed Li ions is also a key factor. Using atomic-level imaging, spectroscopic analysis, and density functional theory (DFT) calculations, we reveal that the mobility of the adsorbed Li ions is much higher on the sGDY@MXene surface compared to that on the pristine MXene surface. Due to the stereostructure of the large TFSI^−^, the high mobility allows Li ions on sGDY@MXene to easily move to optimal positions for cleaving C–S and C–F bonds of the bridged TFSI^−^ (Fig. 1). In contrast, too strong affinity of pristine MXene for Li ions hinders this coordination and cleavage process. As a result, the half-cells and full-cells using the sGDY@MXene-functionalized separators exhibit extended cycle life and enhanced rate performance. This work may provide a general guideline for understanding and regulating the dipole-induced decomposition of fluorinated anions to promote a LiF-rich SEI in LMBs.

## Experimental Section

### Synthesis of Ti_3_C_2_T_x_ MXene

Ti_3_C_2_T_x_ nanosheets were synthesized by the MILD methods. Firstly, 0.5 g of MAX (Ti_3_AlC_2_) was slowly added to a solution containing 10 mL 9.0 M HCl and 0.8 g LiF, and further stirred at 40 °C for 24 h. Then, the mixture was washed with deionized (DI) water and centrifuged at 3500 rpm for 5 min several times until the pH value was about 7. After ultrasonically treated at − 5 °C for 1 h and freeze-dried for 48 h, black and fluffy Ti_3_C_2_T_x_ nanosheets were collected.

### Synthesis of sGDY@MXene Heterojunctions

The GDY precursor of hexaethynylbenzene (HEB) was firstly synthesized through the synthesis of hexakis[(trimethylsilyl)ethynyl]benzene (HEB-TMS) monomers and subsequent deprotection of the trimethylsilyl group, referred to previous publication [37]. Then, sGDY@MXene heterojunctions were fabricated by in situ Glaser–Hay coupling polymerization of HEB on MXene. Specifically, 20 mg of MXene was dispersed in 120 mL of pyridine in the addition of Cu foil as catalyst. Then, the HEB/pyridine precursor was added and stirred in dark at 60 °C for 15 h under the protection of Ar atmosphere, endowing the in situ growth of GDY on the surface of MXene nanosheets. Finally, the product was washed sequentially with acetone, DMF, 1 M HCl, and DI water until the pH value was about 7, and freeze-dried for 48 h to obtain sGDY@MXene heterojunctions.

### Fabrication of sGDY@MXene (or MXene)-Functionalized Separators

Ten milligram of sGDY@MXene or MXene were dispersed in 20 mL of N,N-dimethylformamide (DMF) with 1 mg PVDF and then ultrasonically treated with ice bath for 1 h. Then, the homogeneous dispersion was vacuum filtered on commercial PP separators of Celgard 2500. After vacuum dried at 60 °C for over 24 h, the composite membranes were then cut into discs with diameter of 19 mm and stored in glove box for use as functionalized separators.

### Materials Characterization

The XRD-6100 diffractometer was used with Cu Kα radiation to record the X-ray diffraction (XRD) patterns. The morphology and microstructure of the materials were characterized using scanning electron microscopy (SEM, SU8020) and transmission electron microscopy (TEM, FEI TalosF200S). X-ray photoelectron spectra (XPS, ES CALAB 250Xi) were collected to investigate the chemical states of the surface. The thickness of sGDY@MXene and MXene were examined by the atomic force microscope (AFM, Dimension FastScan Bio). Fourier transform infrared spectroscopy (FTIR, Excalibur HE 3100) was used to identify the functional groups. Raman spectroscopy (LabRAM HR Evo) was also used to determine the bonding structure of sGDY@MXene.

### Cryo-Transmission Electron Microscopic (Cryo-TEM) Investigation

The microstructure of the LiF-rich SEI was investigated by a Thermo Scientific Glacios 2 cryo-TEM equipped with automatic injection system of frozen sample. A 400 mesh Cu grid was applied as the current collector for Li plating/stripping, and assembled in a coin cell equipped with the sGDY@MXene-functionalized PP separator and Li anode. The cell was conducted a plating/stripping process for 50 cycles at a current density of 1 mA cm^−2^ with areal capacity of 1 mAh cm^−2^. Then, the cell was disassembled, and the grid was washed with DME solvent and dried for investigation.

### Characterization of Li-Ion Mobility at the Surfaces of sGDY@MXene and MXene

^7^Li SS-NMR spectra were recorded on Bruker AVANCE III 400WB (Bruker Corporation, Zurich, Switzerland). Samples were prepared by immersing 50 mg sGDY@MXene or MXene in 10 mL solution of 1 M LiTFSI in dimethoxyethane (DME), followed by vacuum infiltration and freeze-drying. Polyethylene oxide (PEO)-based solid polymer electrolytes (SPEs) were fabricated by a typical solution casting method. Specifically, a mixture containing PEO, LiTFSI (with a [EO] to [Li^+^] ratio of 16:1) and 5 wt% fillers (sGDY@MXene or MXene) was dispersed in acetonitrile to form a uniform slurry. Then, the electrolyte slurry was casted in a polytetrafluoroethylene mold and vacuum dried for 12 h at 60 °C to obtain the dry SPE membranes for ionic conductivity test. Electrochemical impedance spectroscopy (EIS) was conducted on symmetric cells, sandwiching the SPE in two stainless steel plates as blocking electrodes. The ionic conductivity (*σ*) was calculated based on the equation: $\sigma = \frac{L}{RS}$, where the *L* is the thickness of the electrolyte membrane, *R* is the resistance of the cell obtained from the EIS data, and *S* is the area of the stainless steel.

### Electrochemical Measurements

Neware electrochemical test system was used to carry out the galvanostatic charge–discharge experiment. All the electrochemical tests were performed on 2032-type coin cells at room temperature of 25 °C, which were assembled in an Ar-filled glove box with water and oxygen content below 0.1 ppm. The Li–Cu half-cells and Li–Li symmetric cells were tested in the electrolytes containing 1.0 M lithium bis(trifluoromethanesulfonyl)imide (LiTFSI) in 1,3-dioxolane (DOL) and dimethoxyethane (DME) (DOL/DME, 1:1 by volume) with 2 wt% LiNO_3_ additive. For the Coulombic efficiency test, Li-Cu half-cells were firstly activated at 0.01–1.0 V for five cycles at 0.05 mA cm^−2^ to stabilize the solid electrolyte interphase (SEI). Then, specific capacities of Li were plated onto Cu electrodes and then stripped with a cutoff voltage of 1.5 V. Li–Li symmetric cells were cycled with the areal capacities of 0.5, 1, and 3 mAh cm^−2^ at the current densities of 0.5, 1, and 3 mA cm^−2^, respectively. The Li-LFP full-cells were tested in the electrolyte containing 1 M LiPF_6_ in EC and DEC (EC/DEC, 1:1 by volume). Each cell contains 60 μL of the electrolyte. The LFP cathodes were made up of 80 wt% LFP, 10 wt% PVDF binder, and 10 wt% Super P with the areal capacity of about 1.58 mAh cm^−2^. To pair with the LFP cathodes, plated Li anodes with an areal capacity of 5.53 mAh cm^−2^ were used, leading to a N/P ratio (capacity ratio of negative electrode to the positive electrode) of 3.5. The Li-LFP full-cells were performed over 2.5–3.8 V. The EIS were tested on the Biologic electrochemical workstation by applying a sine wave with amplitude of 10 mV over frequencies from 100 kHz to 0.1 Hz.

### Computation Methods

All the first-principles calculations were performed by using the Vienna Ab initio Simulation Package (VASP) [38, 39]. The generalized gradient approximation (GGA) in the Perdew–Burke–Ernzerhof (PBE) form and a cutoff energy of 480 eV for plane wave basis set were adopted [40]. A 3 × 3 × 1 gamma-centered [41] k-point was used for sampling the Brillouin zones at structure calculation, whereas a denser mesh of 6 × 6 × 1 was used for the electronic structure calculations. The ion–electron interactions were described by the projector augmented wave (PAW) method [42]. The van der Waals corrections were taken with the semiempirical Grimme parameter DFT-D3 correction [43]. The convergence criteria of structure optimization were chosen to be the maximum force on each atom less than 0.03 eV Å^−1^ with an energy change less than 1 × 10^−5^ eV. To exclude the interactions between the models and their periodic images, a vacuum region of ~ 15 Å is in *c* direction.

### Ab Initio Molecular Dynamics (AIMD) Simulations

The Nosé–Hoover thermostat was used to run all the AIMD simulations in the canonical ensemble (NVT), which has a constant number of atoms, volume, and temperature [44, 45]. Newton’s equations of motion were integrated using Verlet algorithm in the velocity form with a time step of 0.5 fs at 300 K for all the structures. 7 DOL, 5 DME, and 1 LiTFSI molecules were packed in the vacuum space randomly to simulate the electrolyte environment. The number of solvent molecules was evaluated by using the density of liquid solvent (1.140 g cm^−3^ for DOL/DME). For data analysis and molecular visualizations, the application VMD [46] and Materials Studio were utilized.

## Results and Discussion

### Single-Layer GDY on MXene 2D Heterostructured Materials

**Fig. 2:**
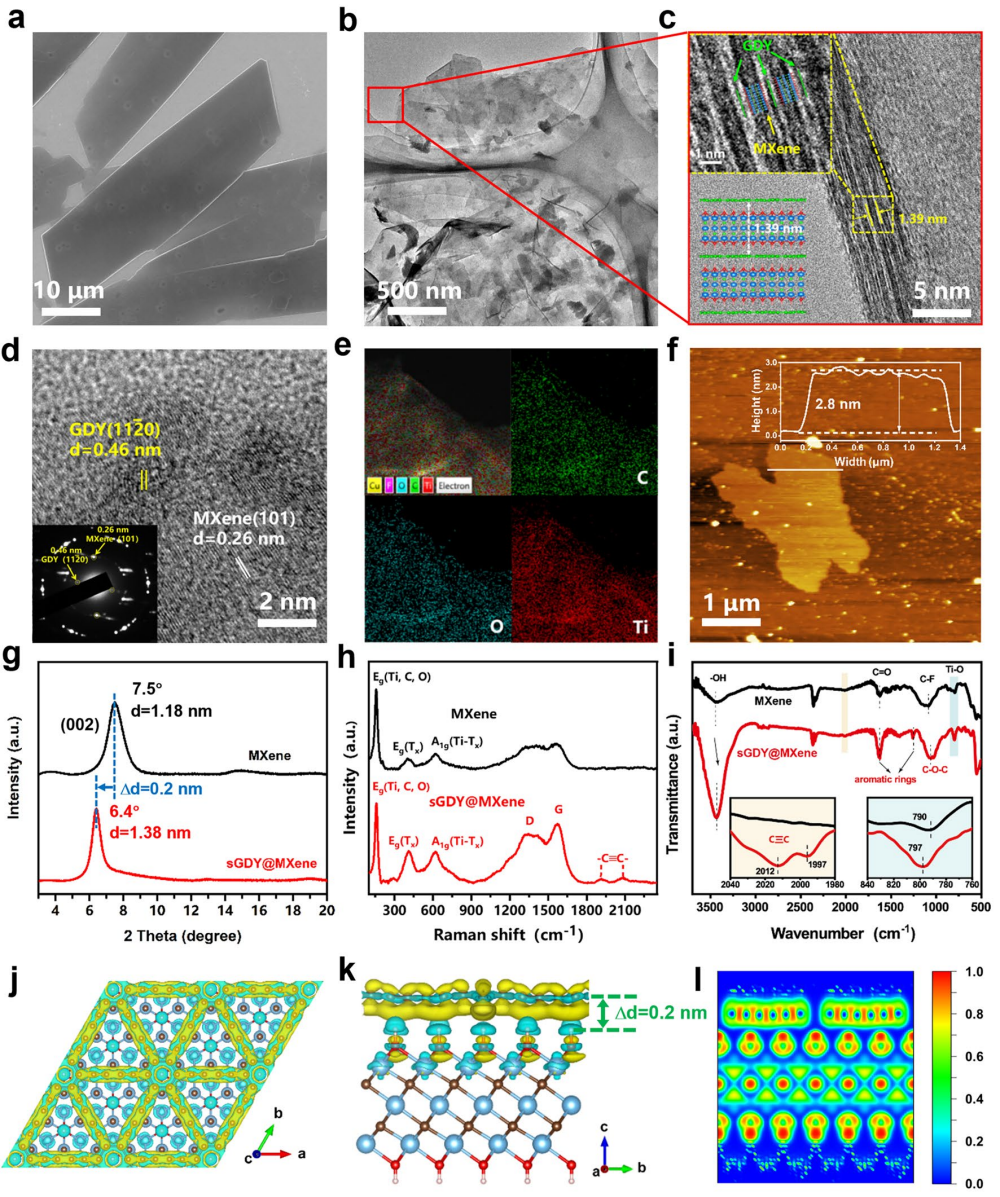
Characterization of sGDY@MXene 2D heterostructured materials. **a**-**e** SEM (**a**), TEM (**b**), HRTEM of the cross section (**c**) and the planar surface (**d**), and elemental mapping (**e**) of sGDY@MXene. **f** AFM image and thickness determination. **g**-**i** XRD (**g**), Raman (**h**), and FTIR spectra (**i**) of sGDY@MXene compared to MXene. **j**, **k** Differential charge density diagrams of sGDY@MXene from the top (**j**) and cross-sectional view (**k**). Light blue: Ti; gray: C; red: O; white: H; yellow: electron-rich; cyan: electron deplete. Isosurface level: 0.0014 |e| Bohr^−3^. **l** Electron localization function (ELF) of the cross section of sGDY@MXene

The fabrication of MXene-based heterostructured materials using self-assembled methods [47, 48] is difficult to control the thickness of GDY on MXene nanosheets. Apart from previous studies, in this work, 2D sGDY@MXene heterostructured materials were synthesized via an in situ Glaser–Hay cross-coupling reaction that occurred directly on MXene flakes with the aid of electrostatically absorbed Cu^2+^ cations, which served as a graphdiyne-coupled catalyst (Fig. S1). Since the catalyst of Cu^2+^ cations was adsorbed and confined only to the very top surface of MXene nanosheets, a single layer of GDY can be carefully tuned within 15 h of synthesis. The morphology and microstructure of the synthesized sGDY@MXene were firstly inspected by SEM and TEM. As shown in Fig. 2a, flakes with lateral sizes in the tens of microns were obtained, the morphology of which is highly consistent with that of the exfoliated pristine MXene flakes in Fig. S2. The TEM image of sGDY@MXene in Fig. 2b shows the translucent flakes with curved edges, indicating their thin and flexible features. To clarify the configuration of the heterostructure, the cross section of the flake was inspected. The high-resolution TEM (HRTEM) in Fig. 2c shows the edge of the flake enlarged from the red frame in Fig. 2b. It demonstrates an alternated arrangement of wide and narrow lattice fringes, corresponding to the laminates of MXene and single layers of GDY, respectively. It is interesting to note that single GDY layers have not only been formed on the surface, but also within the interspace of MXene nanosheets, demonstrating that it is the confined space that induces the growth of single-layered GDY. This proves the successful implementation of our original synthesis design. As also shown in the top-left inset of Fig. 2c, the interplanar spacing (*d* spacing) of a repeating unit, consisting of one laminate of MXene sandwiched between two single layers of GDY, has been determined to be 1.39 nm. Comparing with the *d* spacing of 1.17 nm for pristine MXene laminates (Fig. S2b), the increment in the *d* spacing for the heterostructure is only 0.22 nm. This increment is smaller than the *d* spacing of the restacked GDY (~ 0.35 nm) [49]. It indicates that the interfacial interaction between MXene and GDY is stronger than that between GDY multilayers.

The in-plane microstructure was examined further. The HRTEM image in Fig. 2d shows distinct lattice fringes with an interplanar spacing of 0.26 nm, which can be assigned to (101) planes of MXene, while the fringes of 0.46 nm represent (${11}\overline{2}0$) planes of GDY [50, 51]. In addition, the selected area electron diffraction (SAED) pattern in the inset of Fig. 2d clearly shows the diffraction spots of (${11}\overline{2}0$) planes for GDY and (101) planes for MXene. This substantiates the coexistence of GDY and MXene, suggesting a well-coupled heterostructure. The energy-dispersive X-ray spectroscopy (EDS) mappings in Fig. 2e demonstrate the homogeneous distribution of carbon, titanium, and oxygen within the sGDY@MXene flake. Furthermore, from AFM analysis in Fig. 2f, the thickness of the sGDY@MXene flake has been determined to be 2.8 nm. This thickness corresponds well with two repeating units of the abovementioned sGDY/MXene alternating layers, as also illustrated at the bottom-left inset in Fig. 2c. Accordingly, a 2D/2D vertical heterostructured configuration of sGDY@MXene is confirmed.

Spectral characterization was then conducted to identify the chemical bonding and chemical interaction within sGDY@MXene. As shown in XRD in Fig. 2g, the diffraction peak assigned to (002) plane shows a significant downward shift from 7.5° for MXene to 6.4° for sGDY@MXene. It illustrates the increase in the interplanar spacing from 1.18 nm for MXene to 1.38 nm for sGDY@MXene, suggesting the expansion of 0.2 nm in *d* spacing after the in situ synthesis of single-layered GYD on MXene. This expansion is very close to that obtained in HRTEM analysis in Fig. 2c, confirming the significant interfacial interaction between MXene and GDY. Furthermore, Raman spectra in Fig. 2h present the peaks at 156 and 618 cm^−1^ for both pristine MXene and sGDY@MXene samples, which can be related to the in-plane vibration modes of the MXene flakes [52]. In sGDY@MXene, however, in addition to the apparent D band at 1350 cm^−1^ and G band at 1571 cm^−1^, new doublets appear at 1912 and 2085 cm^−1^, which can be assigned to the vibration of the conjugated diyne chains (–C≡C–C≡C–) as also shown in bare GDY (Fig. S3), substantiating the bonding structure of sGDY on MXene [53, 54]. XPS analysis also verifies the existence of acetylene bonds by identifying *sp*-C in sGDY@MXene (Fig. S4). Moreover, FTIR was further performed to investigate the functional groups. As displayed in Fig. 2i, new peaks emerged at 1632 and 1149 cm^−1^ for sGDY@MXene can be assigned to the skeletal vibration of the aromatic rings. The stretching vibration of alkyne bonds at 2012 and 1997 cm^−1^, as shown in the left inset in Fig. 2i, also confirms the chemical bonding of sGDY [49]. It is noted that the stretching vibration of –OH at 3200–3600 cm^−1^ and the absorption peak of Ti–O at 780–800 cm^−1^ can be observed in both pristine MXene and sGDY@MXene samples. In particular, the –OH band is more attenuated for sGDY@MXene, implying that it should be Ti–OH bonds that interact with sGDY in the construction of the heterostructure. More importantly, the absorption peak from Ti–O bond in sGDY@MXene shows a blueshift, and that from –OH stretching mode shows a redshift, when compared with those in pristine MXene. It implies that electron transfer occurs from the surface group of –OH to sGDY, thereby weakening the –OH bonds while enhancing the strength of Ti–O bonds. This may explain the significant interfacial interaction between MXene and GDY.

The interfacial interaction between sGDY and MXene, as well as the electronic structure of sGDY@MXene has been further studied using DFT calculations. As suggested by the materials characterization results in Fig. 2c–i, a Ti_3_C_2_(OH)_2_ MXene was used to build the model of sGDY@MXene heterostructure. After optimization, it is found that the lattice parameters of *a* and *b* increase by only 1.0% for MXene and decrease by only 1.7% for GDY in sGDY@MXene (Fig. S5 and Table S1), suggesting the successful establishment of the heterostructure. The top and side views of the differential charge density diagrams of the optimized sGDY@MXene are depicted in Fig. 2j, k, respectively. The increased electron density can be clearly seen along the conjugated diyne chains (–C≡C–C≡C–) in sGDY (Fig. 2j). In addition, it is shown from the side view in Fig. 2k that electron transfer occurs from the surface group of –OH to the conjugated diyne chains in sGDY, leading to an increase in O–H bond lengths from 0.973 to 0.988 Å and a decrease in Ti–O bond lengths from 2.183 to 2.159 Å (Table S1). This result corresponds well with the FTIR results in Fig. 2i. Furthermore, Fig. 2k shows that the distance between the plane of sGDY and the plane of H atoms is approximately 0.2 nm. Considering that the van der Waals (vdW) space of ~ 0.2 nm also exist in the stacked MXene nanosheets [55, 56], it can explain the observed 0.2 nm *d* spacing expansion for sGDY@MXene in both XRD and HRTEM analyses. All these results suggest that the optimized model accurately reflects the heterostructure of the synthesized sGDY@MXene. Moreover, the total density of states (TDOS) shows that GDY is a semiconductor, while MXene and sGDY@MXene are both electrically conductive (Fig. S6). The electron localization function (ELF) diagrams of sGDY@MXene in Figs. 2l and S7 further reveal that strong electron delocalization occurs along the conjugated diyne chains in sGDY. Thus, it has been demonstrated that the sGDY gains electrons from MXene to possess electron-rich regions along the conjugated diyne chains, which also serve as electron-conductive pathways. This interfacial interaction increases electron density and enhances electron delocalization effect, transforming the nature of GDY from semiconductive to conductive. In addition, different from MXene with the closely-arranged electron-rich sites of –OH, the sGDY on the MXene surface also preserves the typical features of GDY with the relatively large electron-deficient triangle holes present (Fig. 2j). Therefore, the assembly of the heterostructure integrates the unique structural features from both sGDY and MXene, beneficial for disclosing the reaction landscape of the dipole-induced decomposition of fluorinated anions, which will be discussed in detail in the next section.

### Lithiophilicity-Induced Uniform Li Deposition?

**Fig. 3:**
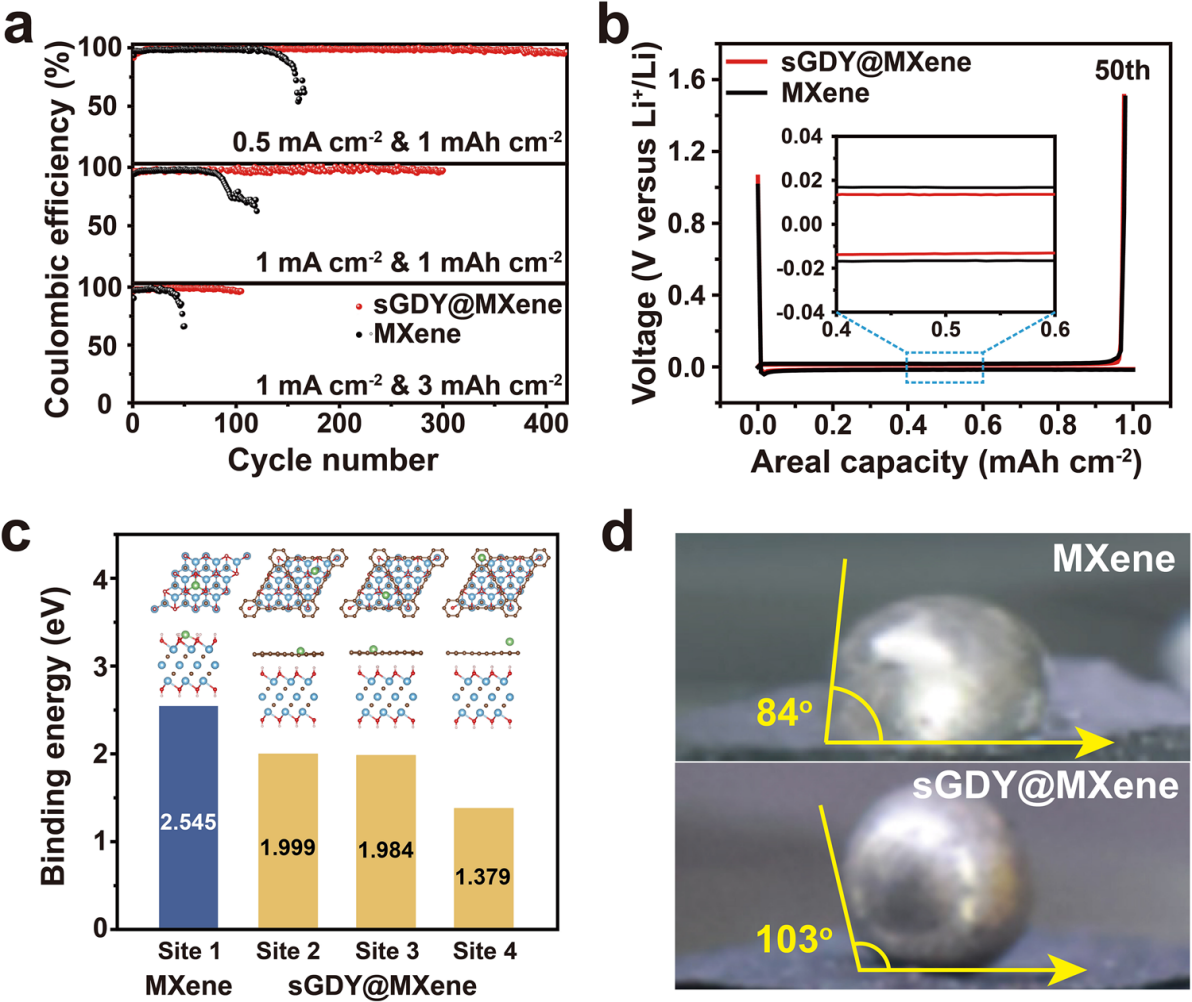
Electrochemical characterization and lithiophilicity of pristine MXene and sGDY@MXene. **a** Coulombic efficiency comparisons at various current densities and areal capacities. **b** Voltage profiles of the 50th plating/stripping cycle at 0.5 mA cm^−2^ for the capacity of 1 mAh cm^−2^. **c** Calculated Li-ion binding energies at different stable sites. **d** Contact angle measurements

To investigate the influence of the as-synthesized functional materials on Li deposition and stripping, sGDY@MXene was loaded onto the commercial PP separator via vacuum filtration. In comparison, pristine MXene-decorated PP separator was also employed. The loading mass of the functional materials was controlled to be ~ 0.5 mg cm^−2^. Figure S8 exhibits the surface morphology of the functional layers, revealing a densely packed arrangement of nano flakes. The cross-sectional images in Fig. S9 indicate that the thickness of the functional layers is about 3 μm. Notably, the sGDY@MXene coating effectively improves the mechanical strength of PP separator as verified by nanoindentation measurements shown in Fig. S10. Galvanostatic Li plating/stripping cycles were tested on Li-Cu half-cells to assess the Coulombic efficiency (CE), with the functional materials facing Cu electrodes. Figure 3a compares CEs of the sGDY@MXene and MXene cells at different current densities and deposition capacities. At a current density of 0.5 mg cm^−2^ and an areal capacity of 1 mAh cm^−2^, the sGDY@MXene cell exhibits excellent cycle stability, maintaining an average CE of 98.5% for over 400 cycles. In comparison, the MXene cell undergoes only ~ 150 cycles before experiencing a serious decay in CE. The voltage profiles of the 50th plating/stripping cycle are depicted in Fig. 3b. As shown in the plateaus displayed in the inset of Fig. 3b, the sGDY@MXene cell shows a smaller voltage hysteresis than that of the MXene cell, indicating a lower overpotential for sGDY@MXene in facilitating Li plating and stripping. When increasing the current density to 1 mA cm^−2^ while keeping the capacity at 1 mAh cm^−2^, the sGDY@MXene cell demonstrates reversible plating/stripping behavior with a high CE of 98.2% for 300 cycles. In contrast, the MXene cell only endures 80 cycles and then experiences a rapid failure under the same test conditions. Even when cycled with a high areal capacity of 3 mAh cm^−2^ at 1 mA cm^−2^, the sGDY@MXene cell still maintains more than 100 cycles with a high CE of 97.8%. In contrast, the CE fluctuates after 30 cycles and then declines precipitously after 40 cycles in the case of the MXene cell. The voltage profiles of the selected cycles (30th to 50th cycles) reveal almost overlapped plating/stripping curves for the GDY@MXene cell, while the stripping capacity of MXene rapidly decays from 3 to ~ 2 mAh cm^−2^ with a constant plating capacity of 3 mAh cm^−2^ (Fig. S11).

It is reported that MXene can facilitate uniform Li deposition due to the presence of abundant lithiophilic sites with a high affinity for adsorbing Li ions [30]. To illustrate this, we conducted DFT calculations about the binding energies of Li ions on both pristine MXene and sGDY@MXene (Fig. 3c). It is shown that the binding energy is the highest at Site 1 on MXene (2.55 eV). In comparison, the binding energies of Li ions at Site 2 and Site 3 (near the edges of triangle holes) of sGDY@MXene are 2.00 and 1.98 eV, respectively, while the binding energy on top of the aromatic ring (Site 4) is 1.38 eV. Thus, although the electron-rich surface would also provide sGDY@MXene with a high affinity for adsorbing Li ions, pristine MXene demonstrates higher lithiophilicty than sGDY@MXene. The contact angle measurements in Fig. 3d, collected by placing a molten liquid Li droplet (at 200 °C in Ar-filled glove box) on the sGDY@MXene or the pristine MXene substrates, also confirm our calculated results. The contact angle of a molten liquid Li droplet on a pristine MXene membrane is 85°, which is lower than that on a sGDY@MXene membrane (103°). Based on the mechanism of lithiophilicity-induced uniform Li deposition, the MXene cell should have demonstrated better performance than the sGDY@MXene cell; however, this is not the case for the experimental results observed in Fig. 3a, b.

### LiF-Rich SEI-Induced Uniform Li Deposition

**Fig. 4:**
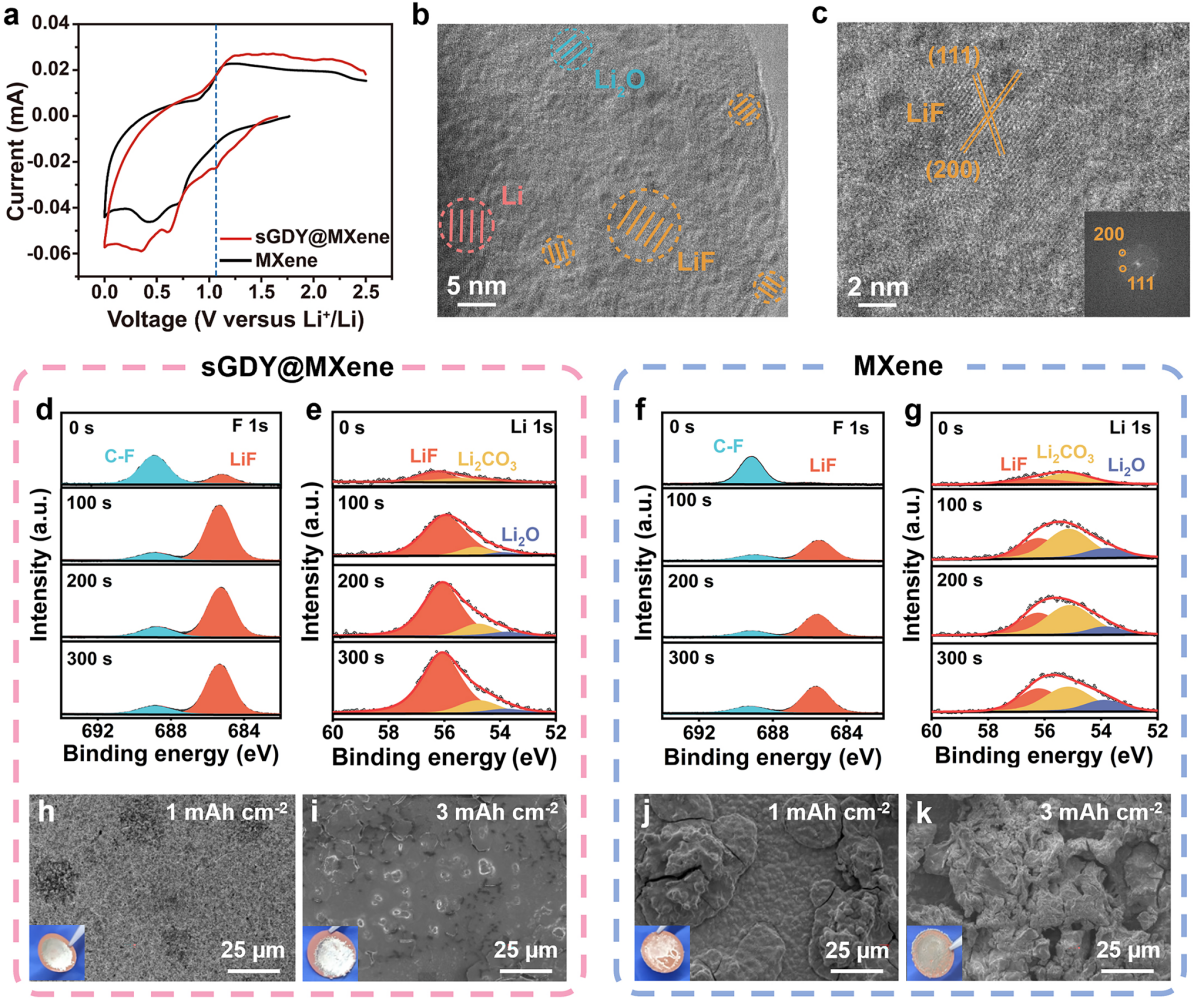
LiF-rich SEI-induced uniform Li deposition. **a** CV curves of the first scan revealing electrochemical decomposition of the electrolyte. **b, c** Cryo-TEM images of the LiF-rich SEI formed in sGDY@MXene cell. **d**-**g** F 1*s* and Li 1*s* XPS depth profiles of the SEI on Li deposits in sGDY@MXene cell (**d**, **e**) and MXene cell (**f**, **g**). **h**–**k** SEM images of the Li deposits on Cu substrates in sGDY@MXene cell (**h**, **i**) and MXene cell (**j**, **k**) with the areal capacities of 1 and 3 mAh cm^−2^, accompanied with the corresponding digital images in the insets

The above results and discussion inspire us to investigate the SEI formed on the lithium-metal surface. Cyclic voltammetry (CV) measurements were conducted to unravel the redox reactions occurring during the process of SEI formation. Upon examining the initial CV scans (Fig. 4a), both pristine MXene and sGDY@MXene cells exhibit cathodic peaks at around 0.4 and 0.6 V, corresponding to the reduction of the solvent [57]. Notably, the reduction potentials for the sGDY@MXene cell are lower than those for the MXene cell, indicating the restriction of the solvent decomposition by sGDY@MXene. More importantly, the sGDY@MXene cell emerges an additional cathodic peak at ~ 1.07 V, which can be associated with the reduction of TFSI^−^ anions as reported previously [19, 21, 58]. This serves as a crucial indicator of the fluorinated-anion-derived transformation, leading to the formation of a favorable LiF-rich SEI.

To confirm the formation of a LiF-rich SEI induced by sGDY@MXene, we have investigated the SEI on the surface of the Li metal anodes at sub-angstrom resolution using cryo-transmission electron microscopy (cryo-TEM). The SEI in Fig. 4b exhibits a classical mosaic structure that consists of an amorphous phase and embedded Li, Li_2_O, and LiF nanocrystals. The calibrated interplanar spacing of 2.43 Å well matches the (110) plane of metallic Li, and that of 2.69 Å well matches the (111) plane of Li_2_O (Fig. S12). More notably, LiF nanoparticles with the lattices corresponding to the (111) and (200) planes can be clearly detected in the SEI (Fig. 4c). To further characterize the chemistry of the SEI, we performed ex situ XPS analysis on the Li deposits after 50 cycles for both sGDY@MXene and pristine MXene cells. As shown in Fig. 4d, the F 1*s* can be deconvoluted into two components, namely C–F at 688.7 eV and LiF at 684.8 eV, which correspond to the intermediate and final products of the TFSI^−^ anion decomposition, respectively. LiF appears at the surface of heterostructure-derived SEI, and with increasing etching depth, LiF becomes the dominant component. Conversely, the LiF signal is absent at the surface of MXene-derived SEI, with a small amount present in the inner part of the SEI (Fig. 4f). Moreover, the Li 1*s* spectra also reveal a considerable LiF content at 56.1 eV in the heterostructure-derived SEI (Fig. 4e). This contrasts with the predominant Li_2_CO_3_ observed at 55.1 eV in MXene-derived SEI (Fig. 4g), which is a characteristic SEI component resulting from the organic solvent decomposition. Figures S13 and S14 also indicate a substantial TFSI^−^ anion decomposition in the sGDY@MXene cell. In addition, the (002) diffraction peak of sGDY@MXene maintains at 6.4° after cell cycling, demonstrating its excellent structural stability as displayed in Fig. S15.

The LiF-rich SEI-induced uniform Li deposition can be clearly seen in Fig. 4h–k, which corresponds well with previous results [10–12, 19]. As displayed in the inset of Fig. 4h, when depositing 1 mAh cm^−2^ of Li, the sGDY@MXene cell produces a shiny metallic surface with Li evenly coated on the Cu substrate. The corresponding SEM image reveals a flat and compact deposition morphology of Li. In contrast, as shown in the inset of Fig. 4j, the MXene cell results in a macroscopically uneven plated layer. The corresponding SEM image shows discrete Li islands in the tens of microns dispersed on the substrate. Figure 4i and Fig. S16a further show that, increasing the plating capacity to 2 and 3 mAh cm^−2^, the Li deposits maintain the smooth and homogeneous morphology in the sGDY@MXene cell, and the surfaces in the optical images remain as shiny as smelted Li foil. Conversely, in the MXene cell under the same conditions, the roughness increases with the increasing deposition capacity (Figs. 4k and S16b), and irregular massive deposits with numerous voids can be observed at 3 mAh cm^−2^. Cross sections of the Li deposits are also inspected. As displayed in Fig. S17, the flat and thin lithium facilitated by sGDY@MXene, with a thickness (17.35 µm) close to the theoretical value (14.5 µm), further confirms the effectiveness of the LiF-rich SEI in suppressing the volume change of deposited lithium. The uniform Li deposition without dendrite formation should be attributed to the LiF-rich SEI, which possesses high mechanical strength to suppress dendrite growth, rapid Li-ion diffusion kinetics to homogenize Li-ion flux, and wide band gap to inhibit electron tunneling and continuous decomposition of the electrolyte [12–16].

All these results suggest that both sGDY@MXene and pristine MXene have high tendency to adsorb Li ions; however, the LiF-rich SEI was only induced by sGDY@MXene. If the adsorbed Li ions are crucial for TFSI^−^ decomposition, why is a LiF-rich SEI not induced by pristine MXene, which have stronger dipole moments.

### Li-Ion Dynamic Bridges to Expedite Dipole-Induced Anion Decomposition

**Fig. 5:**
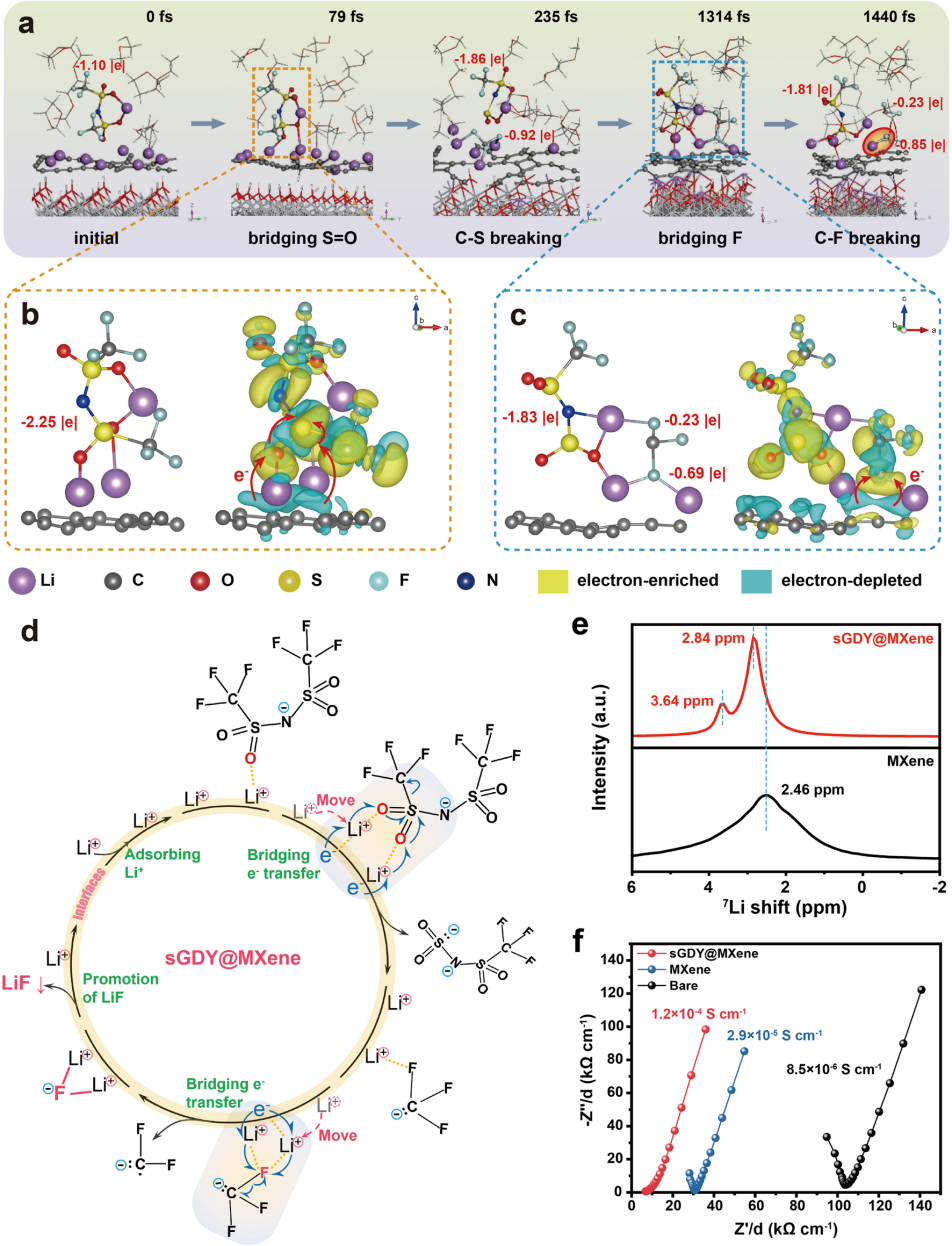
Mechanism of dipole-induced anion decomposition on sGDY@MXene. **a** Snapshots of AIMD simulations at 0, 79, 235, 1314, and 1440 fs revealing the evolution of TFSI^−^ decomposition. **b, c** Li-ion-bridged configurations of TFSI^−^ and the corresponding differential charge density diagrams at 79 fs (**b**) and 1314 fs (**c**). Isosurface level: 0.0017 |e| Bohr^−3^. The instantaneous Bader charges for the anion and its derivatives are in the unit of charge (|e|), illustrating the decomposition dynamics. **d** The proposed reaction pathway of the dynamic Li-ion bridges in expediting TFSI^−^ decomposition on sGDY@MXene. **e**, **f** NMR (**e**) and ionic conductivity (**f**) tests, indicating the high Li-ion mobility on the surface of sGDY@MXene

To address this question, Ab initio molecular dynamics (AIMD) calculations were carried out first to understand the influence of the adsorbed Li ions on the TFSI^−^ decomposition reactions at both sGDY@MXene and pristine MXene surfaces. Without the adsorbed Li ions on sGDY@MXene, it is shown that no TFSI^−^ anion decomposition occurs for 10 ps (Fig. S18). It suggests that, although the surface of sGDY@MXene is electron-rich, the dipoles on the surface are not capable of inducing the anion decomposition by themselves. However, in the case of Li ions adsorbed on sGDY@MXene, the situation is different (Movie S1). As illustrated in Fig. 5a, at 79 fs, two adsorbed Li ions dynamically move to the positions that can coordinate with the two O on O=S=O separately, bridging the TFSI^−^ anion with the sGDY@MXene. The corresponding differential charge density diagram in Fig. 5b clearly illustrates the coordination structure and the charge transfer process at this state. It shows that electrons transfer from the alkyne bond to the two O via the Li-ion bridges, and then further transfer to the S through the two S=O bonds. The Bader charge analysis reveals that approximately − 1.15|e| charge is transferred from sGDY@MXene to the TFSI^−^ anion at 79 fs, leading to the weakening of the C–S bond. At 235 fs, the C–S bond breaks, generating a CF_3_^−^ group. At the same time, the Li ions adjust their positions to coordinate with F on the CF_3_^−^ group, with one Li ion for each –F. Afterward, at 1314 fs, when Li ions move to the positions where two Li coordinate with one F on the CF_3_^−^ group, prominent electron transfer occurs from sGDY@MXene to the coordinated F via the two bridged Li ions (Fig. 5c). The increased electron density around this F elongates and weakens the corresponding C–F bond, ultimately leading to its cleavage. In the final step, LiF formation occurs as indicated by a red circle at 1440 fs in Fig. 5a. Figure 5d summaries the proposed reaction pathway of Li-ion-bridged TFSI^−^ decomposition. Regarding the simulation on the pristine MXene (Fig. S19), although one of the adsorbed Li ions may occasionally coordinate with the TFSI^−^ anion through O on O=S=O or F on –CF_3_, the immobilized Li ions cannot interact collaboratively. As a result, the TFSI^−^ anion does not undergo decomposition but instead moves away from the MXene surface.

According to the simulation results, although Li-ion adsorption is necessary for the anion decomposition reaction, Li-ion mobility on the polar surface is essential for accelerating this reaction due to the stereostructure of the large TFSI^−^ anion. For experimental validation, ^7^Li solid-state nuclear magnetic resonance (SS-NMR) spectra were recorded on sGDY@MXene and pristine MXene after immersion in a 1 M LiTFSI solution, followed by filtration to obtain dry powders for testing. The local coordinated environment and migration dynamics of the adsorbed Li^+^ can be obtained. As depicted in Fig. 5e, the sGDY@MXene shows two ^7^Li peaks at 2.84 and 3.64 ppm, corresponding to two different adsorbed sites (near the edges of triangle holes and on top of the aromatic ring as shown in Fig. 3c). In addition, these two peaks present significant downfield shift compared with the ^7^Li peak at 2.46 ppm in the spectrum for pristine MXene, indicating electron de-shielding environment and suppressed coordination of the Li ions adsorbed on the sGDY@MXene. It reveals that, although Li-ion adsorption is apparent on both surfaces, the adsorption strength is lower on sGDY@MXene than that on pristine MXene. This result is consistent with our calculation results in Fig. 3c and lithiophilicity test in Fig. 3d. More importantly, a single broad signal was observed in the spectrum of pristine MXene, attributed to solid-state dipole–dipole and quadrupolar couplings between ^7^Li sites [59]. In contrast, two narrow signals in the spectrum for sGDY@MXene mean that the adsorbed Li ions are in a more symmetric environment or experiencing rapid motion at room temperature so that the quadrupolar interaction is averaged [60]. This provides clear evidence that the mobility of the adsorbed Li ions on sGDY@MXene has been significantly enhanced compared to that on pristine MXene. To quantitatively compare the mobility of the adsorbed Li ions on the two polar surfaces, we adopt the method used for testing ionic conductivity in solid polymer electrolytes (SPEs) [61]. Polyethylene oxide (PEO)-based SPEs were tested, with sGDY@MXene and pristine MXene added separately as fillers in symmetric cells applying stainless steel plates as blocking electrodes. As shown in Fig. 5f, the ionic conductivities of the sGDY@MXene-incorporated polymer electrolytes are 1.2 × 10^−4^ S cm^−1^, which is over 4 times higher than that of the MXene-incorporated polymer electrolytes (2.9 × 10^−5^ S cm^−1^) and over 14 times higher than that of the bare polymer electrolyte (8.5 × 10^−6^ S cm^−1^). This indicates that the filler-PEO interface supplies additional ionic conductive pathways, with the increased mobility of adsorbed Li ions on the sGDY@MXene surface contributing to the increased ionic conductivity. The interfacial charge transfer kinetics were further investigated employing Li||Li symmetric cells. As verified in Figs. S20 and S21, the significantly increased exchange current density and reduced activation energy demonstrate the effectiveness of sGDY@MXene in promoting interfacial Li-ion transfer kinetics.

**Fig. 6:**
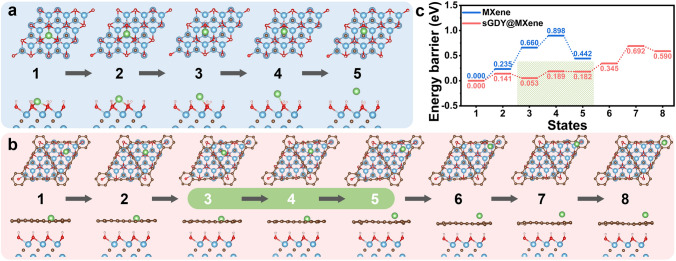
DFT calculations of Li-ion migration barrier. Li-ion migration on the surface of MXene (**a**) and sGDY@MXene (**b**), with the calculated energy barriers at corresponding sites (**c**)

To understand the differences in the mobility of adsorbed Li ions on the sGDY@MXene and pristine MXene, we conducted DFT calculations to determine the energy barrier for Li-ion migration on both surfaces. The migration of the Li ion from the most stable position on the pristine MXene (Site 1 in Fig. 3c) to the loosely-bound position on top of –OH groups in Fig. 6a demonstrates a high energy barrier of 0.898 eV (also indicated by blue steps in Fig. 6c). In contrast, the migration of the Li ion from the most stable position on the sGDY@MXene (Site 2 or Site 3 in Fig. 3c) to the loosely-bound position on top of the aromatic ring (Site 4 in Fig. 3c) in Fig. 6b shows a lower energy barrier of 0.692 eV (also indicated by red steps in Fig. 6c). More importantly, as shown in Fig. 5a and Movie S2, the TFSI^−^ anion can coordinate with Li ions in the green highlighted region (States 3 to 5) in Fig. 6b, c for the sGDY@MXene. Thus, the actually energy barrier for Li-ion migration on the sGDY@MXene before coordinating with the TFSI^−^ anion is as low as 0.189 eV. This is reasonable because sGDY on MXene possesses relatively large triangle holes (0.542 nm) [62], allowing Li ions to easily adjust their positions along and over the hole. These Li-ion dynamic bridges would expedite the coordination and bond cleavage processes between Li ions and TFSI^−^ anions, improving the reaction kinetics. Notably, a significant increase in the LiF content has also been confirmed in the full-cell configurations with the salt of LiPF_6_ used in the electrolyte under the effect of sGDY@MXene (Fig. S22), demonstrating the universality of the conclusion drawn from the LiTFSI example.

### Performance of Cells Containing sGDY@MXene

**Fig. 7:**
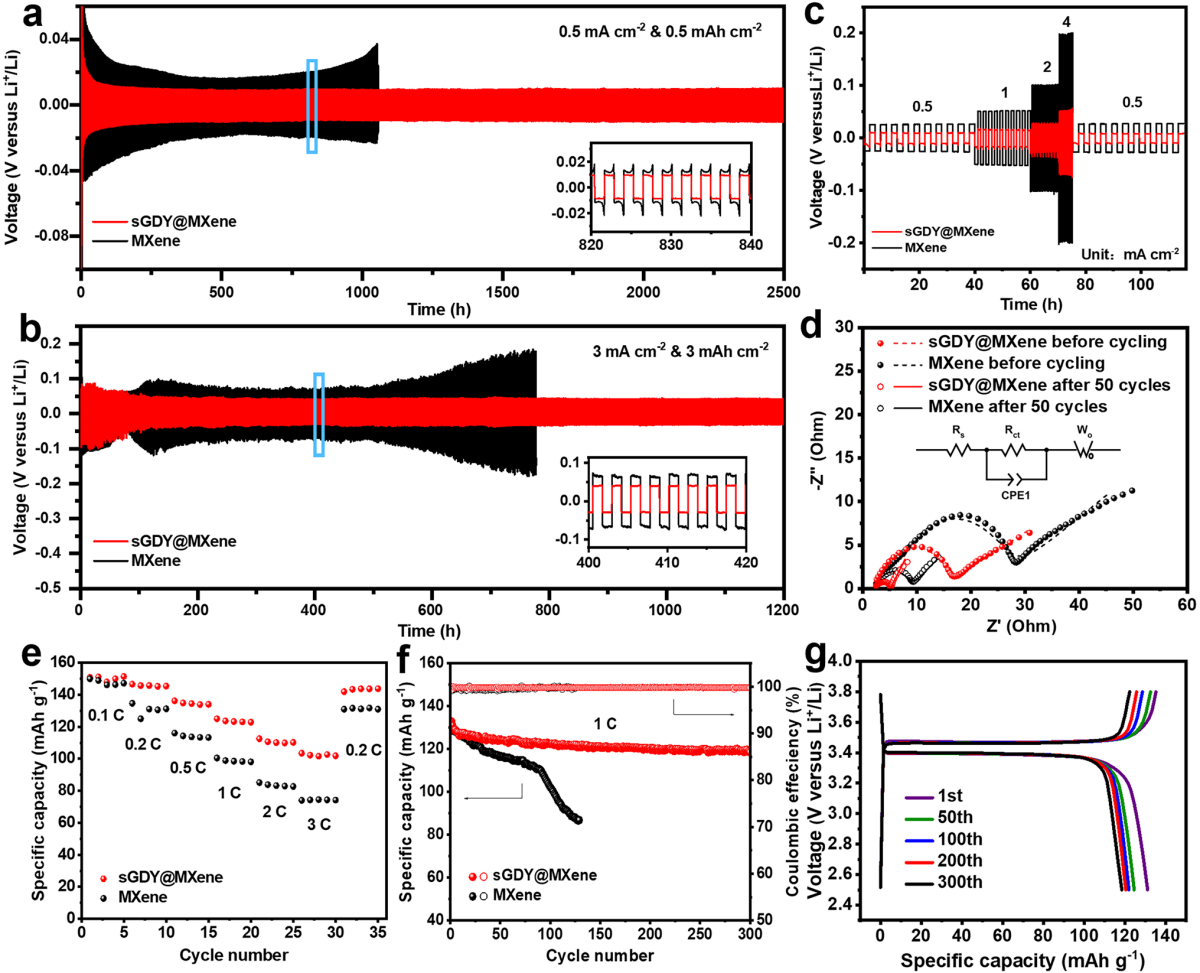
Electrochemical performance of Li–Li symmetric cells and Li-LFP full-cells. **a**, **b** Long-term Li plating/stripping curves of the symmetric cells at 0.5 mA cm^−2^ & 0.5 mAh cm^−2^ (**a**) and 3 mA cm^−2^ & 3 mAh cm^−2^ (**b**). **c** Voltage curves of the symmetric cells at various current densities. **d** EIS and fitting of the symmetric cells before and after 50 cycles (inset shows the equivalent circuit). **e**, **f** Rate performance (**e**) and cyclic stability (**f**) of the Li-LFP full-cells. **g** Voltage profiles of the sGDY@MXene full-cell at different cycle numbers

Due to the delicate balance between lithiophilicity and Li-ion mobility achieved on the polar surface of sGDY@MXene, performance advantages of using sGDY@MXene as the functional layer can be expected at the cell level. As displayed in Fig. 7a, the sGDY@MXene cell performs excellent plating/stripping cyclability for 2500 h at a current density of 0.5 mA cm^−2^ with an areal capacity of 0.5 mAh cm^−2^, maintaining an ultra-stable overpotential at 10 mV. Even under increased current densities and capacities (Figs. 7b and S23), the sGDY@MXene cells still remain outstanding stability, lasting for 1800 h at 1 mA cm^−2^ & 1 mAh cm^−2^, and 1200 h at 3 mA cm^−2^ & 3 mAh cm^−2^. This performance demonstrates uniform Li deposition and highly reversible plating/stripping behaviors. Moreover, the rate behavior of the symmetric sGDY@MXene cell outperforms the MXene cell as depicted in Fig. 7c, exhibiting more stable plating/stripping profiles and lower overpotentials under a specific capacity of 0.5 mAh cm^−2^ at all measured current densities ranging from 0.5 to 4 mA cm^−2^. EIS measurements and corresponding fitting results in Fig. 7d and Table S2 reveal that the sGDY@MXene cell exhibits smaller charge transfer resistance of 12.2 Ω before cycling and 2.2 Ω after 50 cycles, compared to those of the MXene cell (22.5 Ω before cycling and 6.6 Ω after 50 cycles). These results underscore that the LiF-rich SEI facilitates Li-ion transfer across the SEI, promoting reversible plating/stripping kinetics and ensuring uniform Li deposition at high rates for the sGDY@MXene cell.

Li//sGDY@MXene//LiFePO_4_ and Li//MXene//LiFePO_4_ full-cells were also assembled utilizing commercial LiFePO_4_ (LFP) as the cathode and plated Li as the anode. The Li metal anodes were prepared by galvanostatically plating Li on Cu current collectors at a current density of 0.1 mA cm^−2^ until reaching a certain areal capacity with a N/P ratio of 3.5. As displayed in Fig. 7e, the sGDY@MXene full-cell manifests an outstanding rate behavior, outperforming that of the MXene full-cell, particularly at high C rates. Notably, the charge–discharge overpotential of the sGDY@MXene full-cell shows only a small increase from 49 to 136 mV as the C rate increases from 0.1 to 3 (Fig. S24). In contrast, the overpotential of the MXene full-cell increases substantially from 62 to 423 mV as the C rate increases from 0.1 to 3. Moreover, the sGDY@MXene full-cell exhibits excellent cycle stability, as shown in Fig. 7f. It maintains a capacity of 120 mAh g^−1^ after 300 cycles at 1 C, indicating a high capacity retention of 90%. Figure 7g shows that the GDY@MXene full-cell presents the steady charge–discharge profiles with almost unchanged plateaus, outperforming those of the MXene full-cell (Fig. S25). The Li-LFP full-cell exhibits outstanding balanced performance across four key dimensions: N/P ratio, cycle rate, cycle life, and capacity retention. This performance surpasses the full battery data reported in the literature, as illustrated in Fig. S26 [63–68]. The inferior electrochemical performances of pristine PP separator (Figs. S27-S29) and GDY decorated PP (Figs. S30-S32) further highlight the effectiveness of the sGDY@MXene heterostructure in promoting uniform Li plating and stripping cycles. These results confirm the enhanced Li plating/stripping kinetics and long-term stability induced by the favorable LiF-rich SEI at the cell level. The promoted formation of the LiF-rich SEI can be attributed to the dynamic Li-ion bridges on the GDY@MXene, which expedite the kinetics of the dipole-induced TFSI^−^ decomposition reaction, as discussed above.

## Conclusions

In this work, a new insight into dipole-induced anion decomposition has been provided, demonstrated by a novel 2D heterostructured material, sGDY@MXene. sGDY@MXene has been successfully fabricated through the confined-space growth of single-layered GDY on Cu^2+^-adsorbed Ti_3_C_2_-MXene nanosheets. Comprehensive characterizations and simulations reveal that the sGDY gains electrons from MXene to possess electron-rich regions along the conjugated diyne chains, which also serve as electron-conductive pathways. Meanwhile, the sGDY on the MXene surface also preserves the typical features of GDY with the relatively large electron-deficient triangle holes present. By integrating the unique structural features from both sGDY and MXene, we found that, instead of direct electron transfer from surface polar groups to TFSI^−^ anions, the adsorbed Li ions on sGDY@MXene act as dynamic bridges connecting the electron-donating sGDY@MXene to TFSI^−^. Due to the stereostructure of the large TFSI^−^ anion, the adsorbed Li ions have to coordinate collaboratively with the oxygen in O=S=O and the fluorine in the broken –CF_3_^−^ segment. Highly mobile Li ions on the sGDY@MXene facilitate this coordination, accelerating the cleavage of C–S and C–F bonds in the bridged TFSI^−^. In contrast, immobilized Li ions on the more lithiophilic pristine MXene retard these coordination and bond cleavage processes. Under this reaction landscape, a rightful balance between lithiophilicity and Li-ion mobility has been achieved on the polar surface of sGDY@MXene, promoting the formation of the LiF-rich SEI. Thus, it favors uniform Li deposition and rapid Li-ion transfer across the LiF-rich SEI, contributing to the long-term stability of lithium-metal anodes. This work may offer a new perspective for regulating dipole-induced decomposition of large anions in the electrolyte (Li salts or additives) to promote a fast Li-ion conducting SEI, applicable not only to LMBs but also to traditional lithium-ion batteries, sodium-ion batteries, solid-state batteries, and beyond.

## Supplementary Information

Below is the link to the electronic supplementary material.

Supplementary file1 (AVI 85204 KB)

Supplementary file2 (AVI 23629 KB)

Supplementary file3 (DOCX 8011 KB)
