# Supplementary material for: Revisiting Dipole-Induced Fluorinated-Anion Decomposition Reaction for Promoting a LiF-Rich Interphase in Lithium-Metal Batteries
Source: Nanomicro Lett. 2025 Jan 20;17:111. doi: 10.1007/s40820-024-01637-5 (PMC11747066; doi:10.1007/s40820-024-01637-5)
Supplement: Supplementary file 3 — Supplementary file3 (DOCX 8011 KB) [file 40820_2024_1637_MOESM3_ESM.docx]

Supporting Information for

**Revisiting Dipole-Induced Fluorinated-Anion Decomposition Reaction for Promoting a LiF-Rich Interphase in Lithium-Metal Batteries**

Liu Wang^1,†^, Jiahui Guo^1,†^, Qi Qi^2^, Xiaotong Li^2^, Yuanmeng Ge^1^, Haoyi Li^1^, Yunfeng Chao^1^, Jiang Du^2,^*, Xinwei Cui^1,2,3,^*

^1^ Henan Institutes of Advanced Technology, Zhengzhou University, Zhengzhou 450003, P. R. China

^2^ College of Materials Science and Engineering, Zhengzhou University, Zhengzhou 450001, P. R. China

^3^ State Key Laboratory of Coking Coal Resources Green Exploitation, Zhengzhou University, Zhengzhou 450001, P. R. China

^†^ Liu Wang and Jiahui Guo contributed equally to this work.

*Corresponding authors. E-mail: [xinweic@zzu.edu.cn](mailto:xinweic@zzu.edu.cn) (Xinwei Cui); [dj@zzu.edu.cn](mailto:dj@zzu.edu.cn) (Jiang Du)

**Supplementary Figures and Tables**


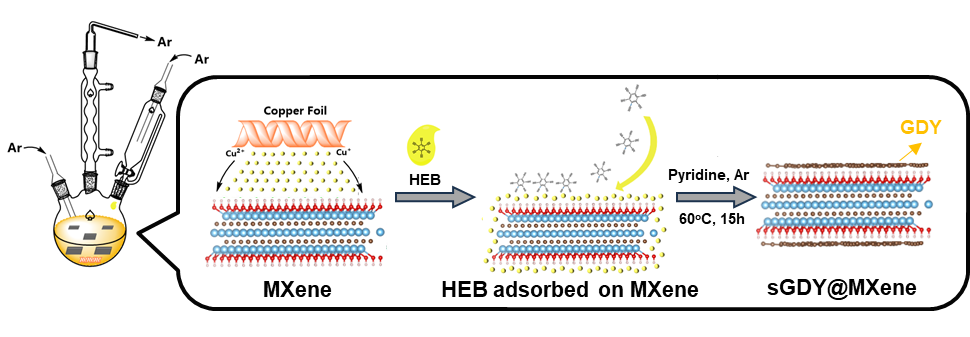


**Fig. S1** Synthesis scheme of sGDY@MXene 2D heterostructured materials


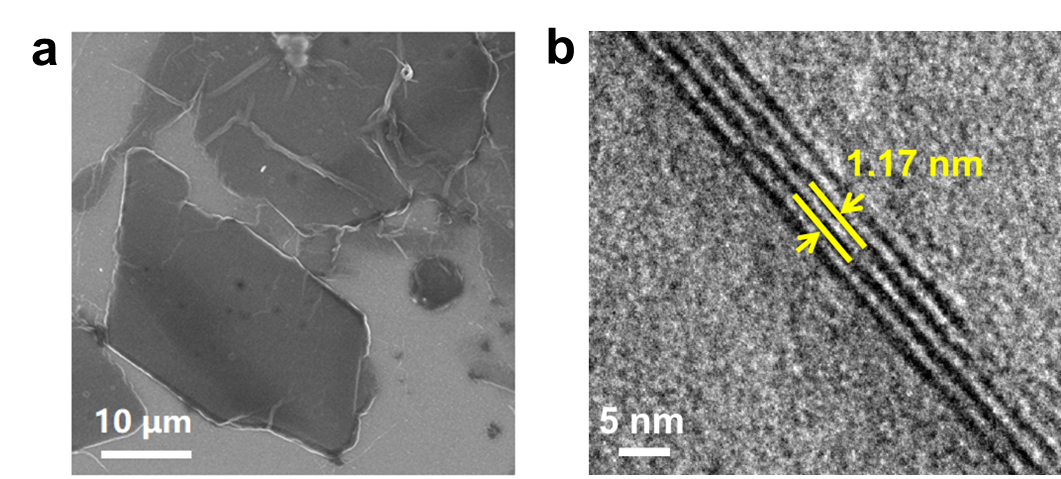


**Fig. S2** (**a**) SEM image and (**b**) HRTEM cross-sectional view of the pristine MXene


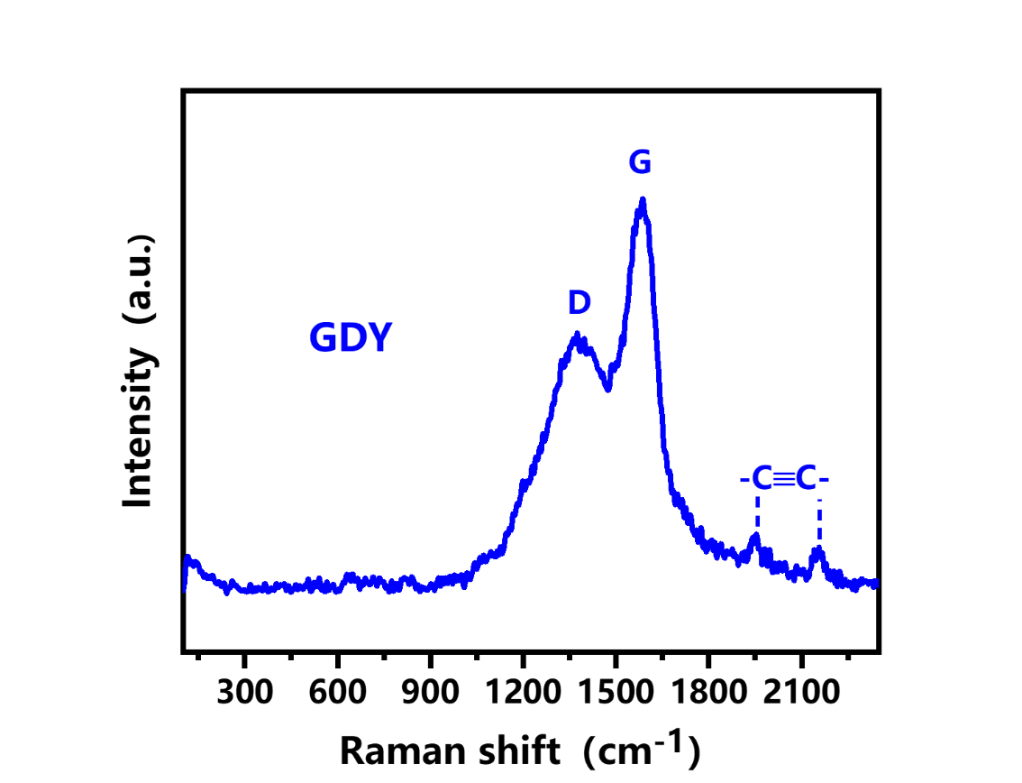


**Fig. S3** Raman spectrum of bare GDY


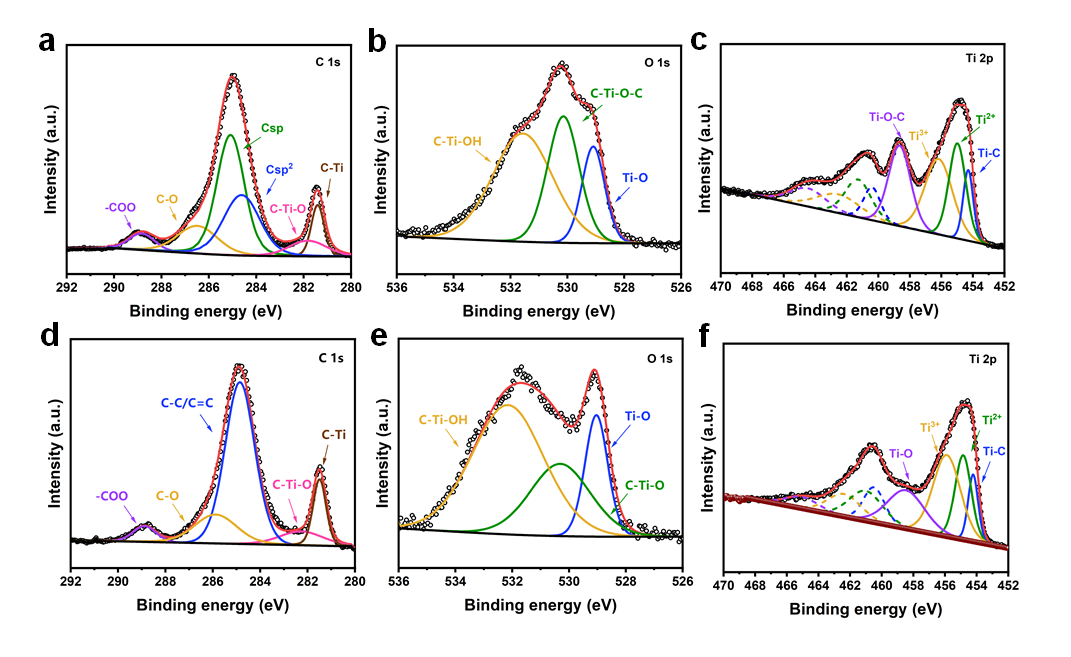


**Fig. S4** XPS analysis of (**a-c**) sGDY@MXene and (**d-f**) MXene


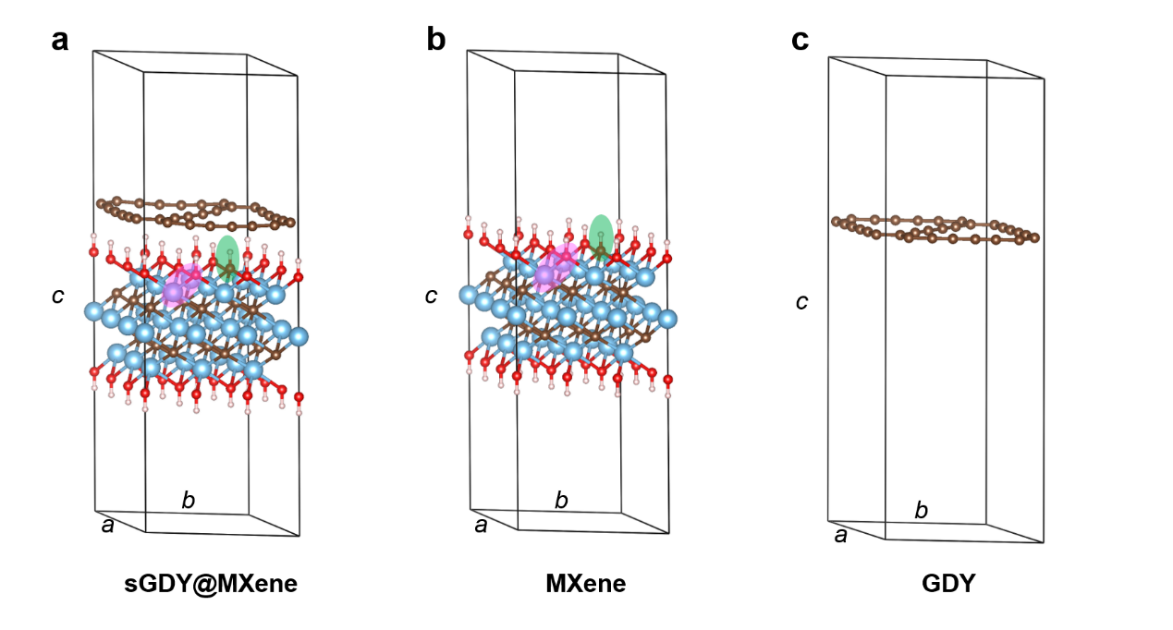


**Fig. S5** Supercells of (**a**) sGDY@MXene, (**b**) MXene, and (**c**) GDY

**Table S1** Lattice parameters of sGDY@MXene, MXene, and GDY, and the bond lengths of Ti−O and O−H

| **Sample** | **a (Å)** | **b (Å)** | **c (Å)** | **Ti−O (Å)** | **O−H (Å)** |
| --- | --- | --- | --- | --- | --- |
| **sGDY@MXene** | 9.293 | 9.292 | 27.637 | 2.159 | 0.988 |
| **MXene** | 9.198 | 9.198 | 27.637 | 2.183 | 0.973 |
| **GDY** | 9.452 | 9.452 | 27.637 |  |  |


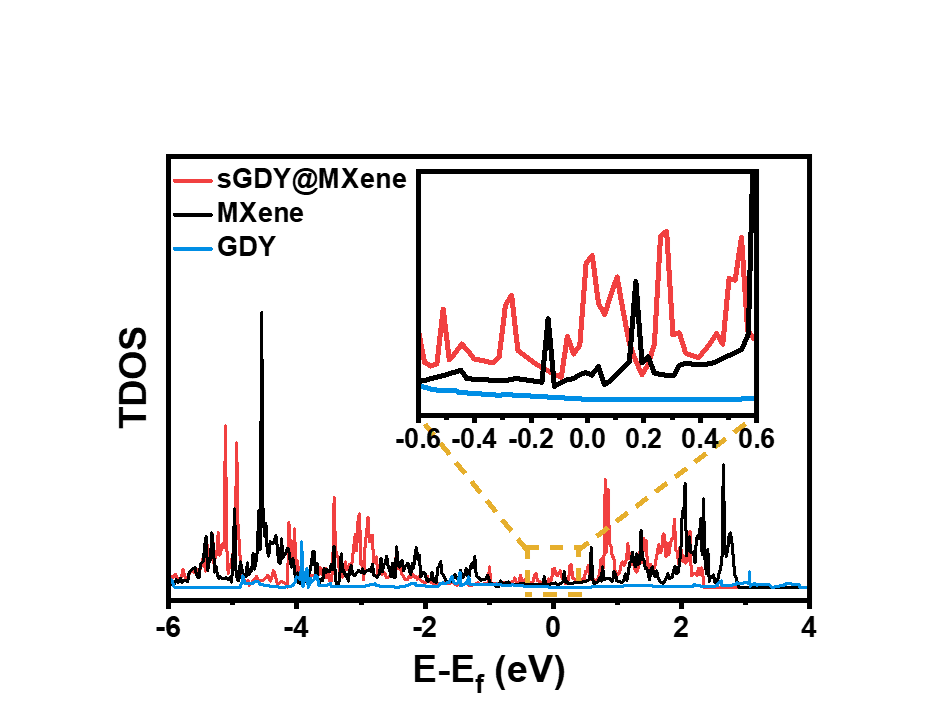


**Fig. S6** TDOS of sGDY@MXene, MXene, and GDY

**
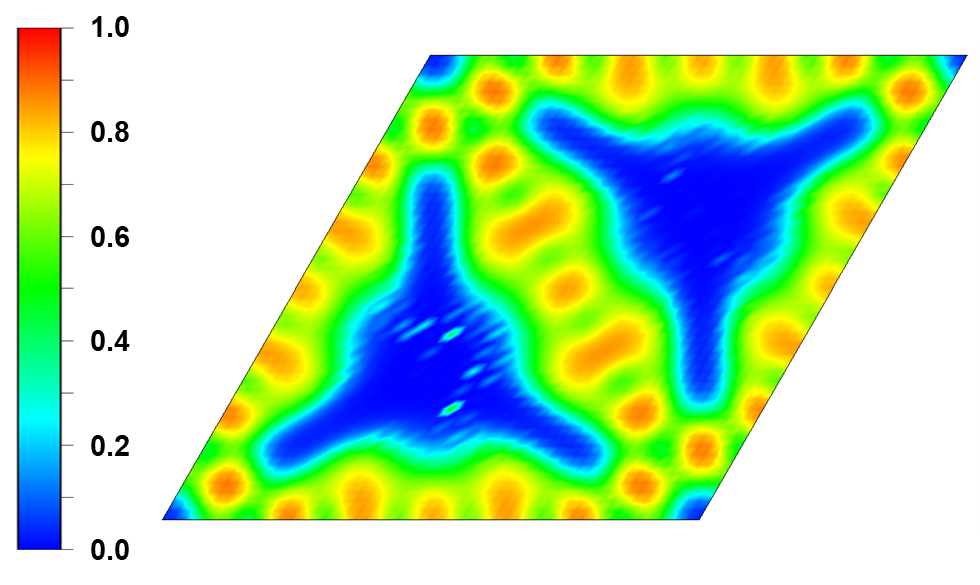
**

**Fig. S7** Top view image of the electron localization function (ELF) of sGDY@MXene


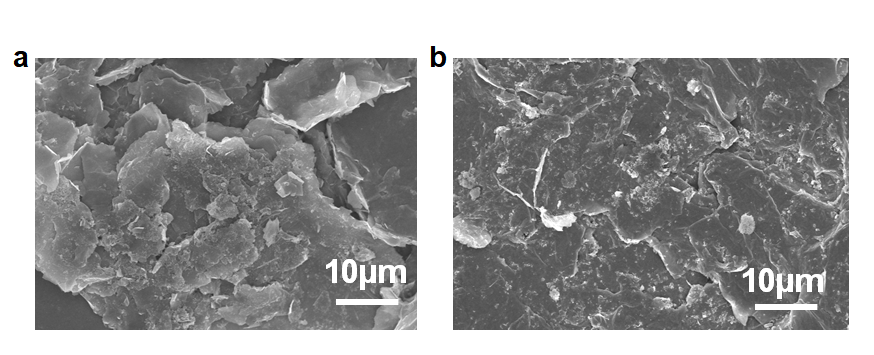


**Fig. S8** SEM images showing the surface of (**a**) sGDY@MXene and (**b**) MXene on PP separators


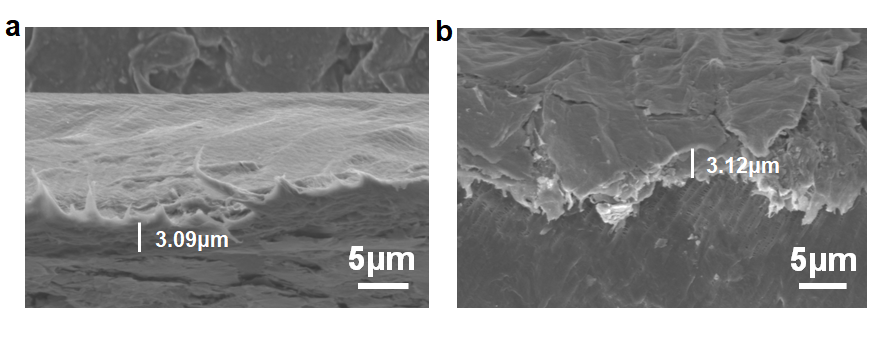


**Fig. S9** Cross-sectional SEM images determining the thickness of (**a**) sGDY@MXene and (**b**) MXene functional layers on PP separators


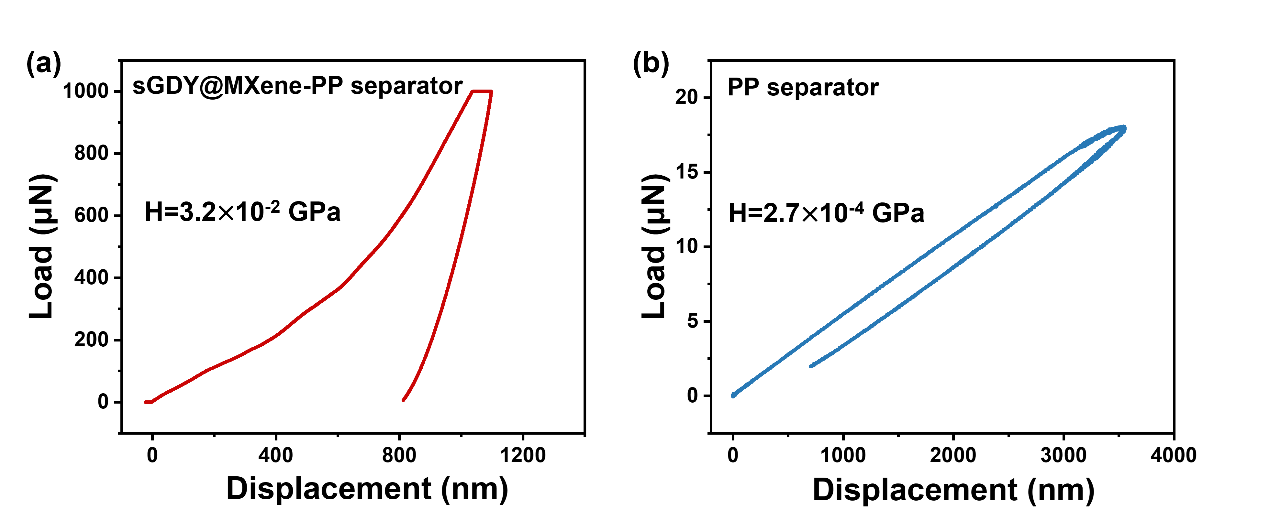


**Fig. S10** Nanoindentation curves of sGDY@MXene-PP (**a**) and pristine PP separator **(b**)


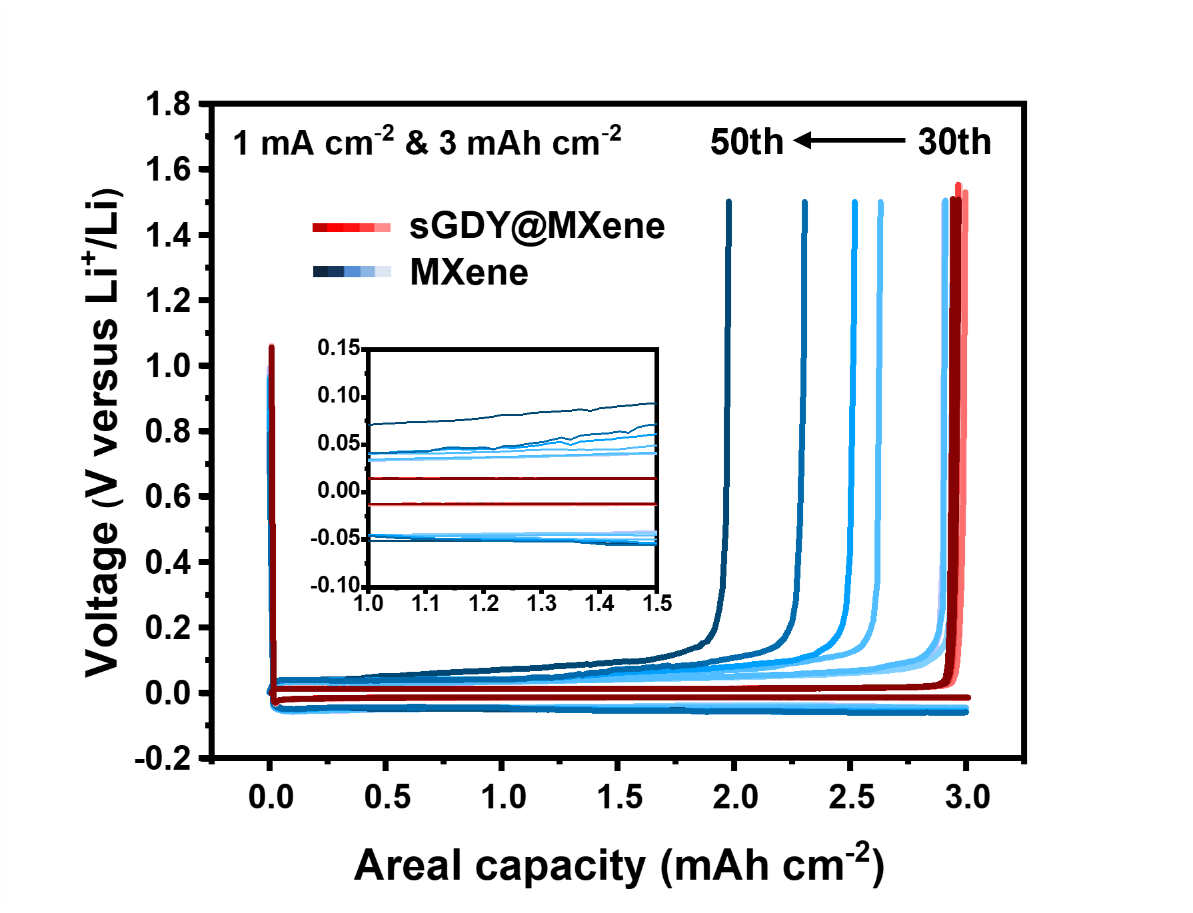


**Fig. S11** Selected plating-stripping curves of Li-Cu cells at the range of 30−50 cycles under 1 mA cm^−2^ for 3 mAh cm^−2^


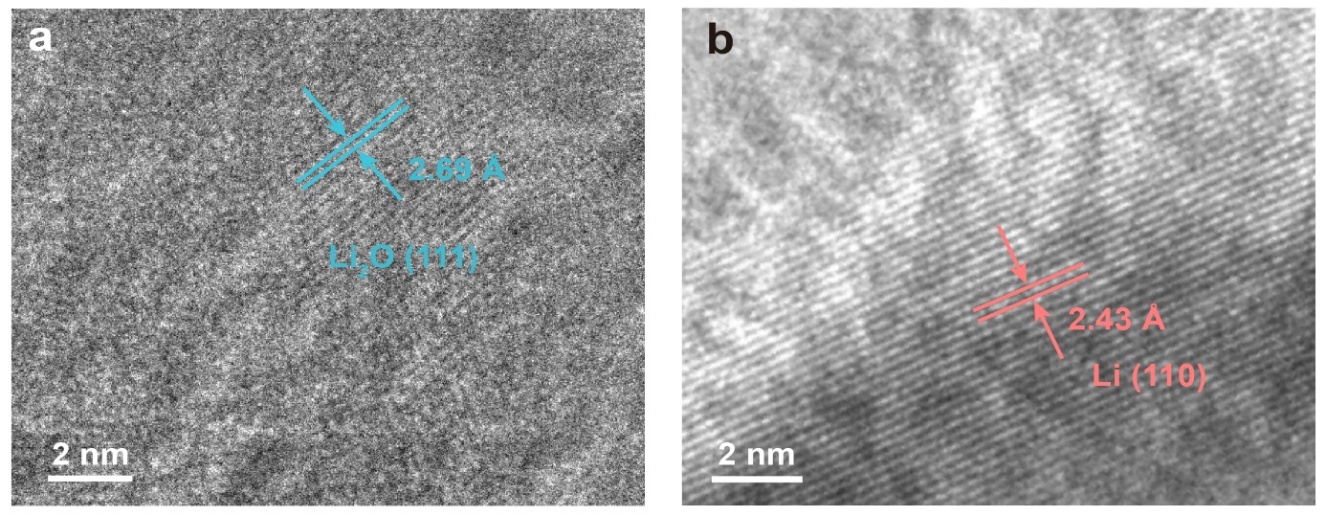


**Fig. S12.** Cryo-HRTEM images of the (**a**) Li_2_O and (**b**) Li regions in the LiF-rich SEI induced by sGDY@MXene


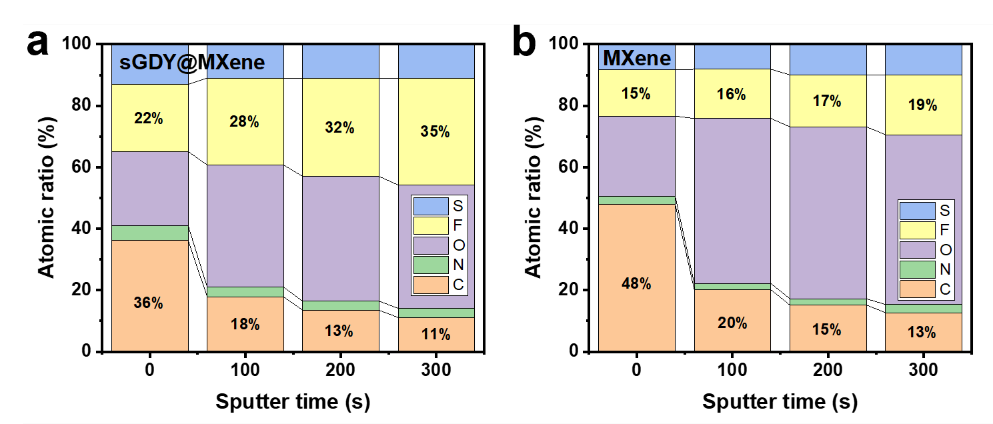


**Fig. S13** Atomic content for non-Li elements of S, F, O, N and C in the SEI of the (**a**) sGDY@MXene cell and (**b**) MXene cell at various sputter times derived from XPS measurements


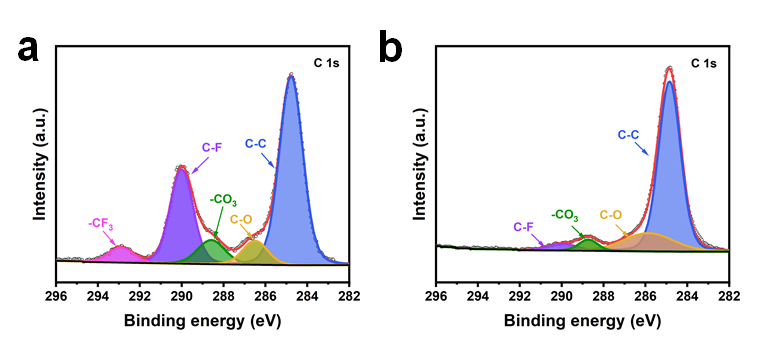


**Fig. S14** High resolution C 1s XPS spectra of the SEI on the deposited Li after 50 cycles in Li-Cu cells for the (**a**) sGDY@MXene and (**b**) MXene cells without Ar-ion sputtering


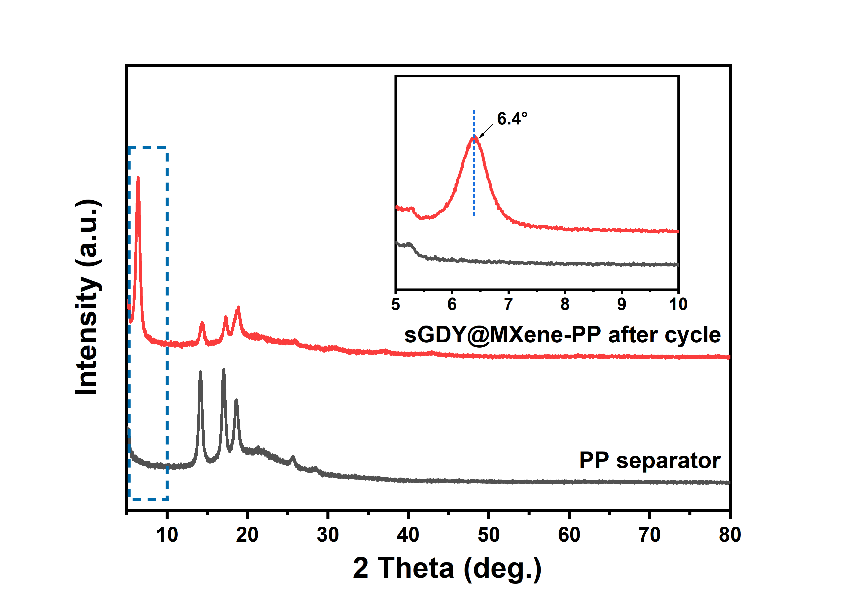


**Fig. S15** XRD pattern of the sGDY@MXene-PP separator after cycling compared with bare PP separator


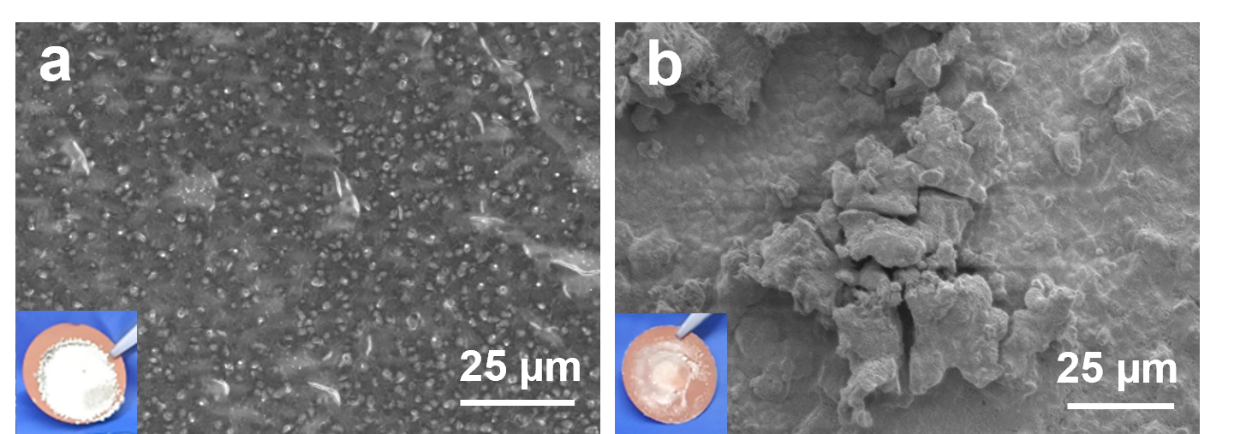


**Fig. S16** SEM images and photographs (insets) of Li deposits on Cu electrodes with the capacity of 2 mAh cm^−2^, using (**a**) sGDY@MXene and (**b**) pristine MXene functionalized PP separators in the Li-Cu cells


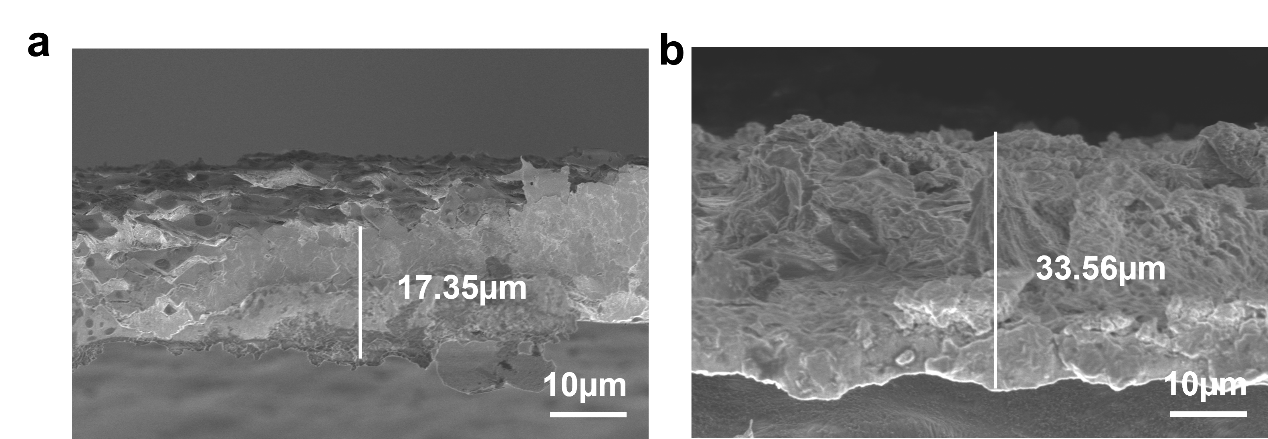


**Fig. S17** Cross-sectional SEM images showing 3 mAh cm^−2^ of Li deposits on Cu, using (**a**) sGDY@MXene and (**b**) pristine MXene functionalized PP separators in the Li-Cu cells


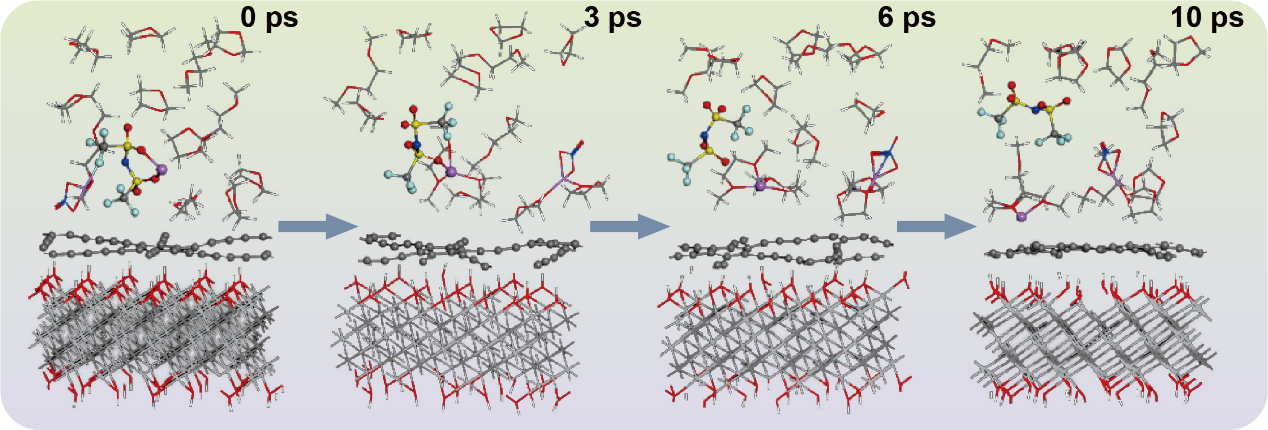


**Fig. S18** Snapshots of AIMD simulations taken without the adsorbed Li ions on sGDY@MXene, and no TFSI^−^ anion decomposition occurs for 10 ps


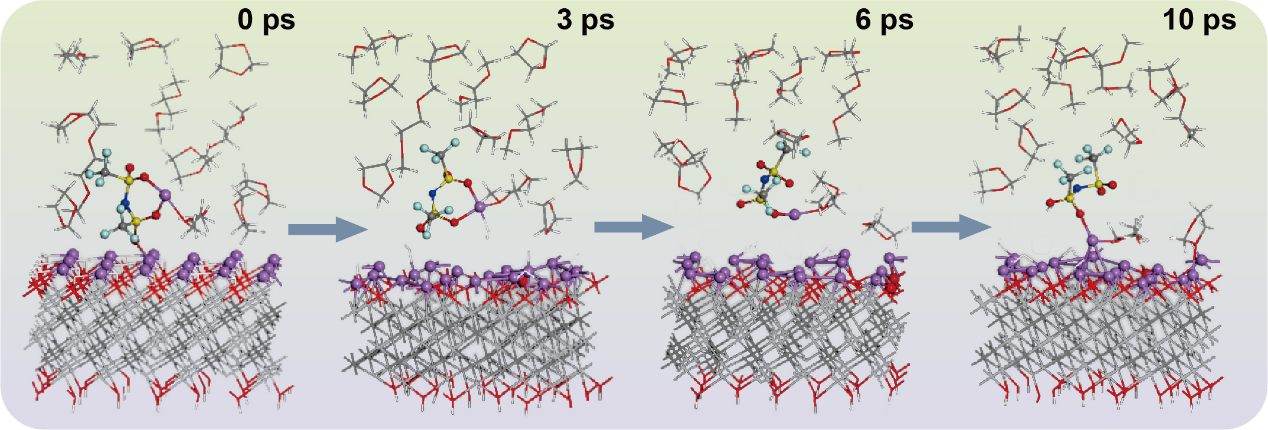


**Fig. S19** Snapshots of AIMD simulations taken with the Li ions adsorbed on pristine MXene, and the TFSI^−^ anion moves away from the MXene surface


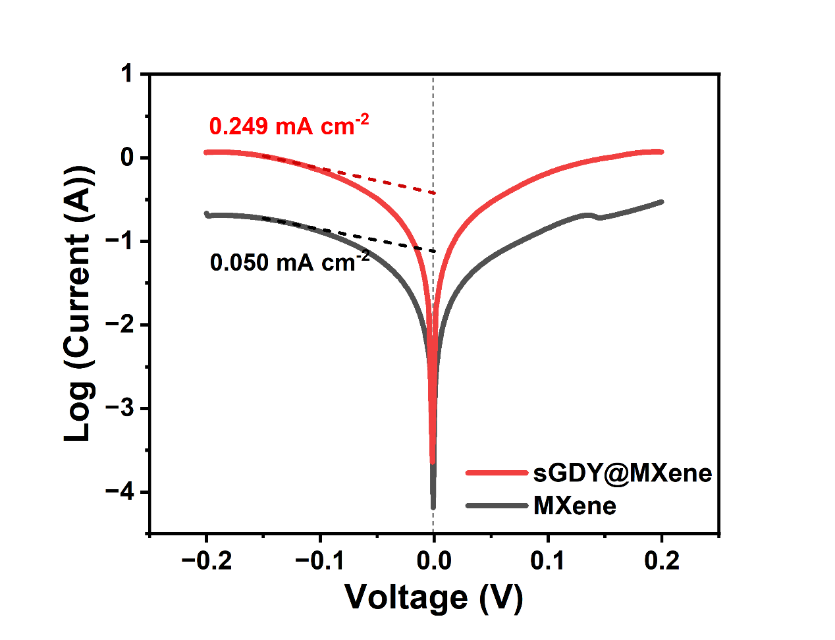


**Fig. S20** Tafel plots showing exchange current densities of Li||Li symmetric cells of sGDY@MXene and MXene


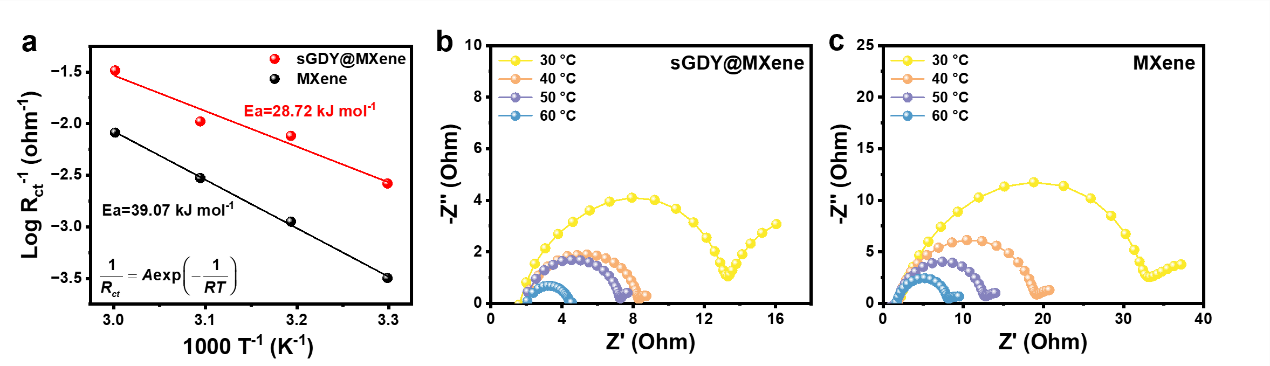


**Fig. S21** (**a**) Arrhenius fitting of activation energy derived from (**b-c**) EIS spectra at various temperature of sGDY@MXene (**b**) and MXene (**c**)


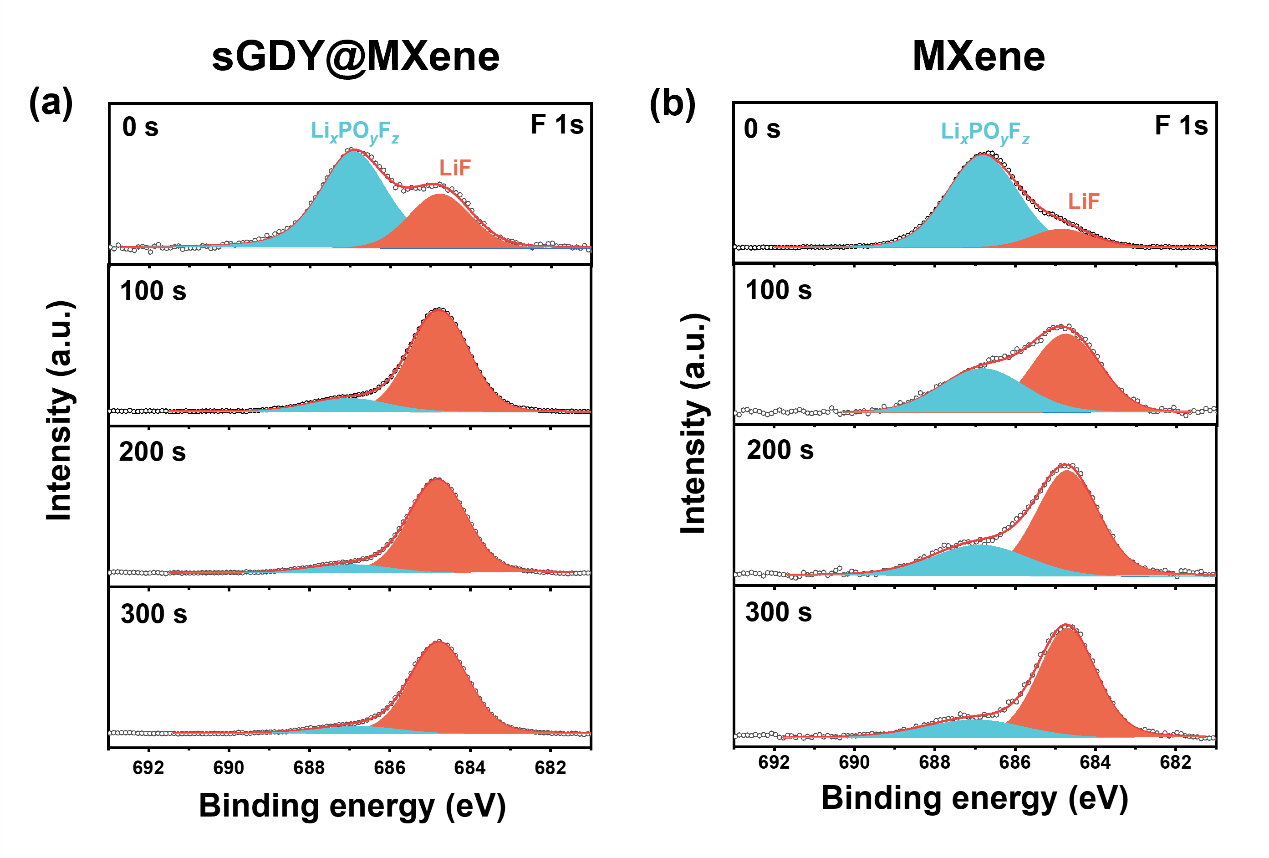


**Fig. S22** F 1s XPS depth profiles of the SEI on Li anode extracted from the full cells of sGDY@MXene (**a**) and MXene (**b**) using LiPF_6_ as the salt in the electrolyte


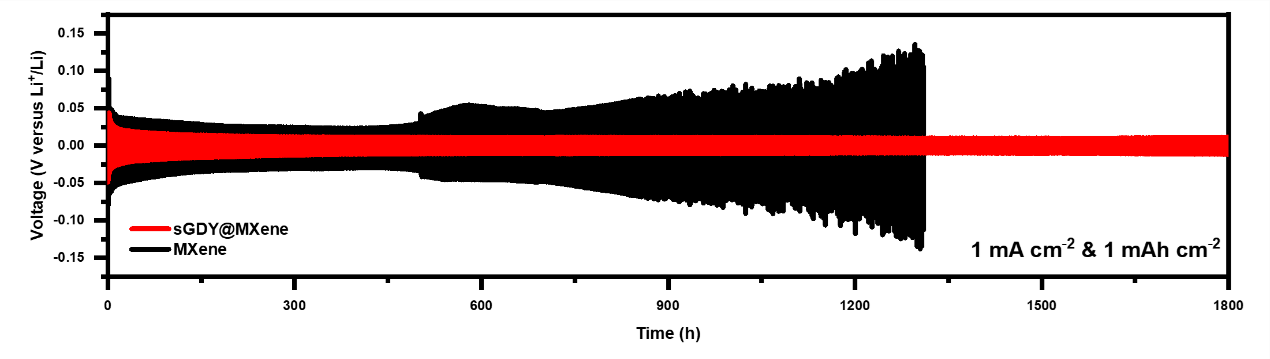


**Fig. S23** Long-term plating/stripping cycling of Li-Li symmetric cells with sGDY@MXene and MXene at 1 mA cm^−2^ with the areal capacity of 1 mAh cm^−2^

**Table S2** Fitting results of EIS before and after cycling

| Samples | Rs (Ω) | Rct (Ω) |
| --- | --- | --- |
| sGDY@MXene before cycling | 1.8 | 12.2 |
| sGDY@MXene after 50 cycles | 2.3 | 2.8 |
| MXene before cycling | 2.1 | 22.5 |
| MXene after 50 cycles | 2.2 | 6.6 |


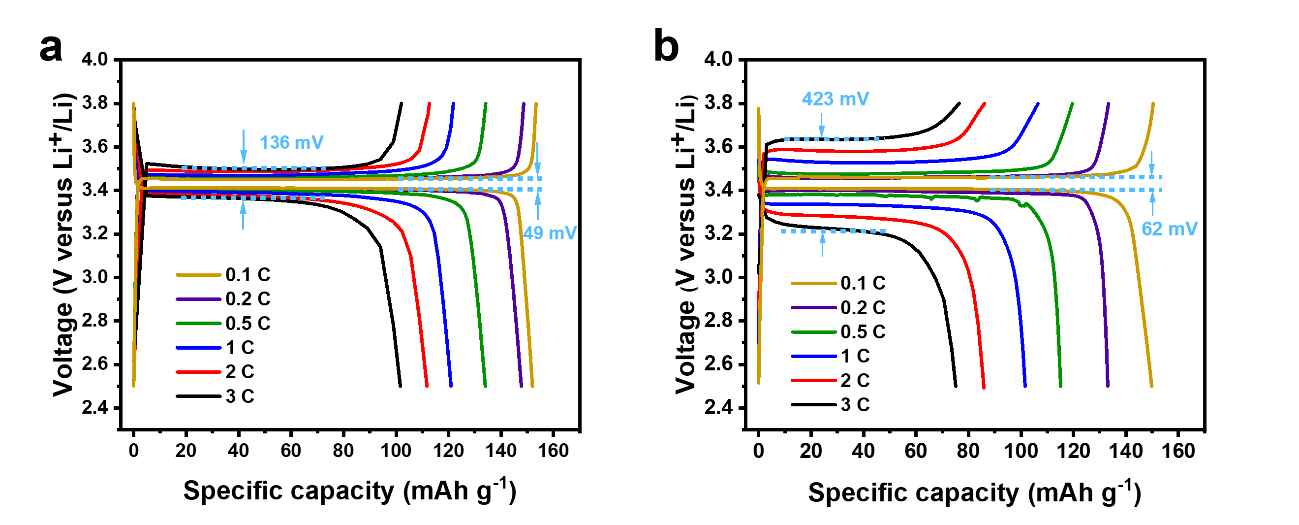


**Fig. S24** Charge-discharge profiles of Li-LFP full cells equipped with (**a**) sGDY@MXene and (**b**) MXene at various C-rates ranging from 0.2 C to 3 C


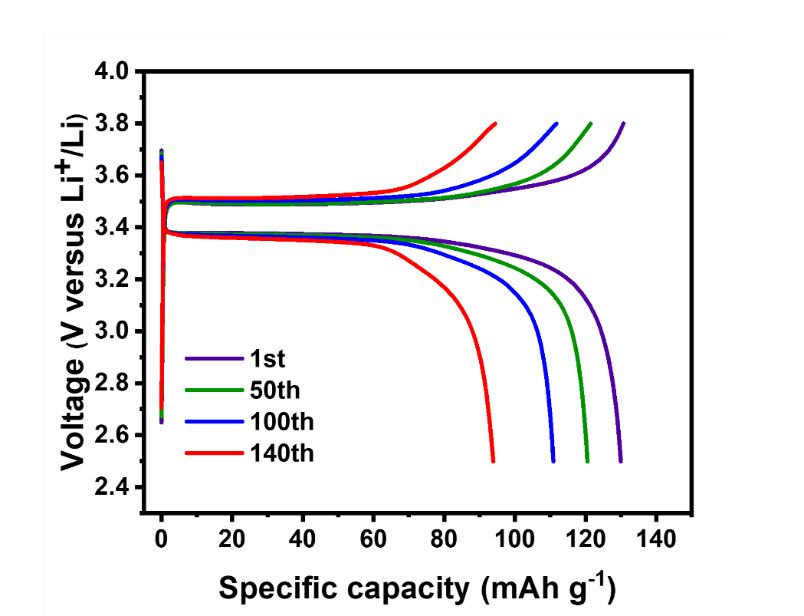


**Fig. S25** Charge-discharge curves of different cycle numbers for the MXene full cell at 1 C


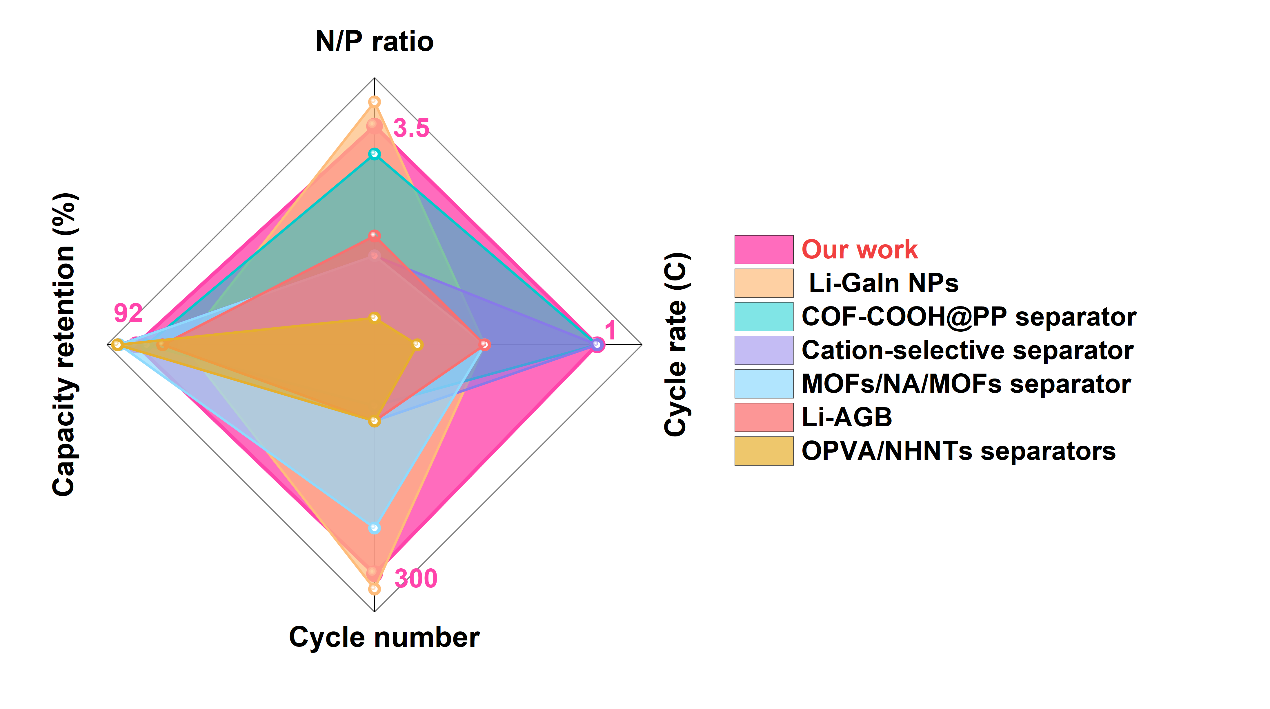


**Fig. S26** Comparison of full cell performance in N/P ratio, cycle rate, cycle number and capacity retention [S1-S6]


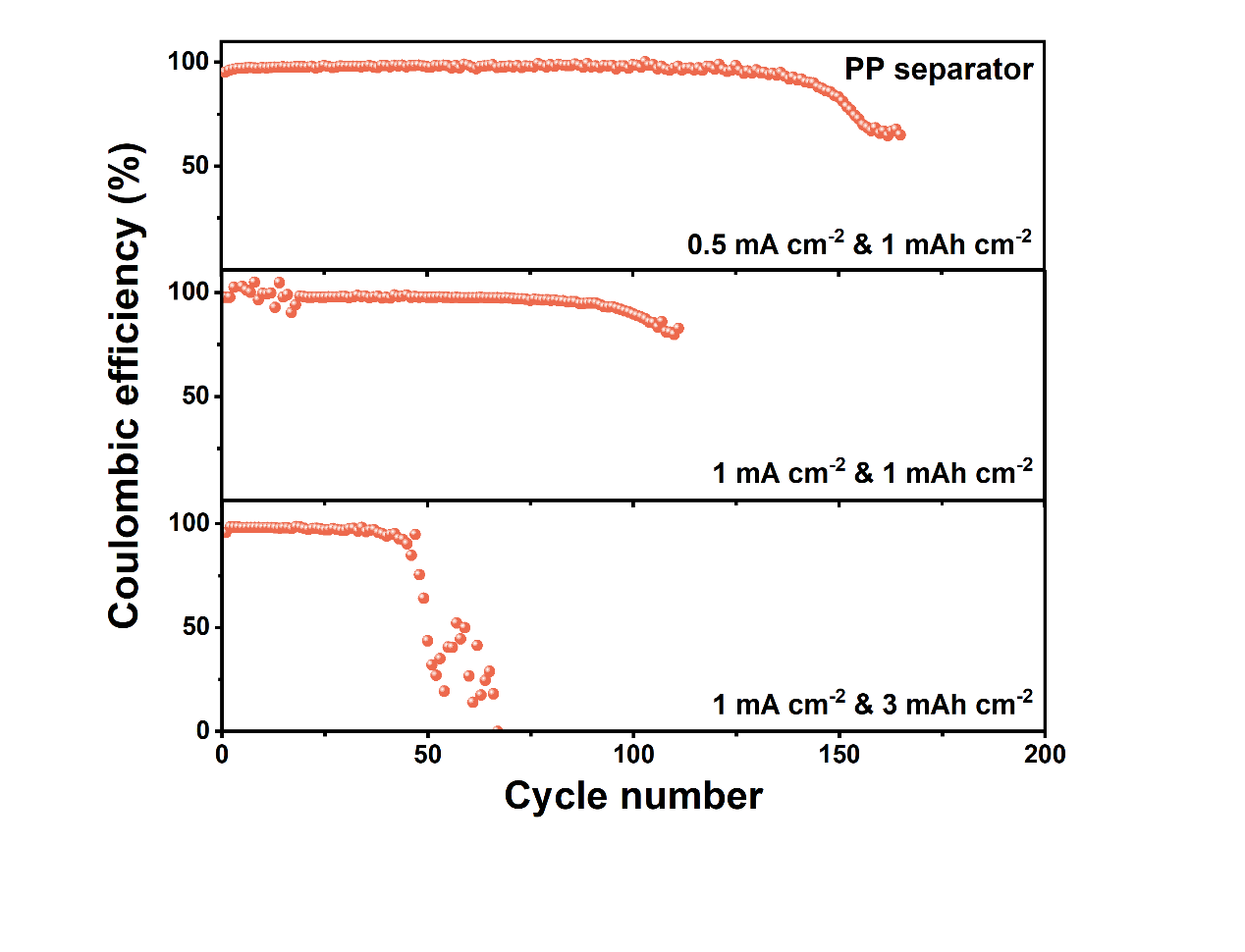


**Fig. S27** Coulombic efficiency of Li-Cu cell equipped with PP separator at various current densities and areal capacities


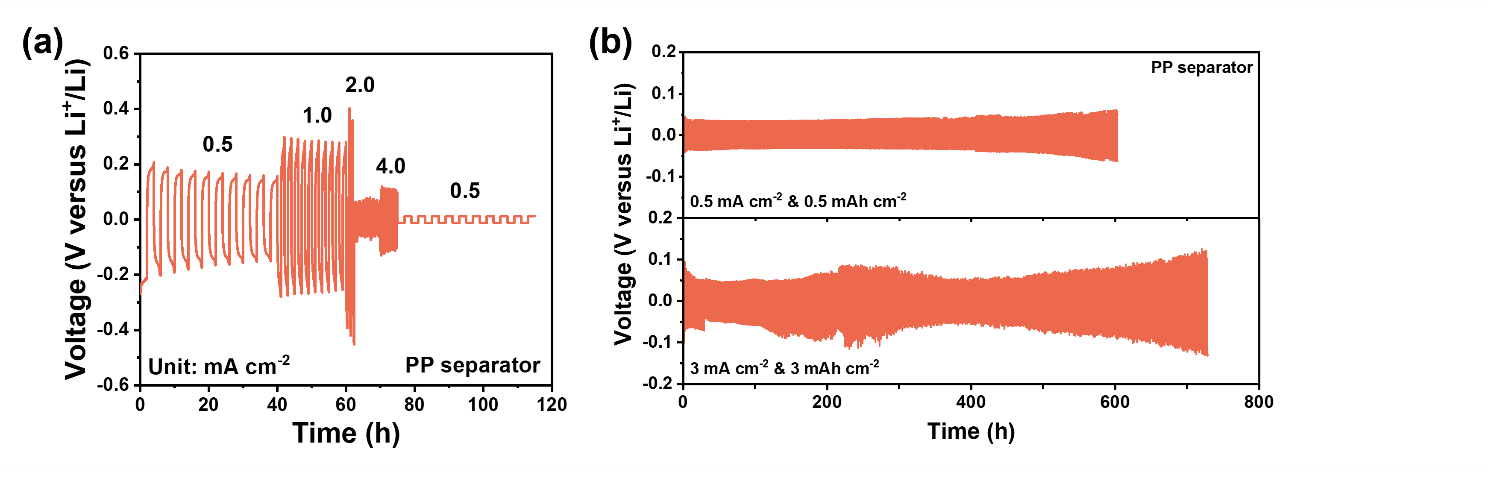


**Fig. S28** Rate (**a**) and cycle (**b**) performances of Li-Li symmetric cells with PP separator


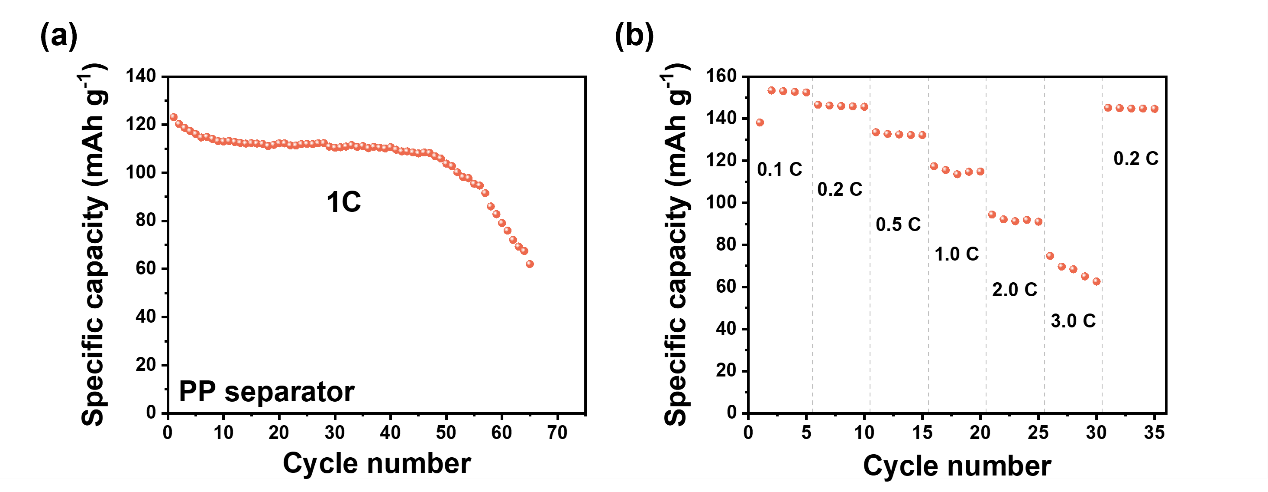


**Fig. S29** Cycle (**a**) and rate (**b**) performances of Li-LFP cells with PP separator


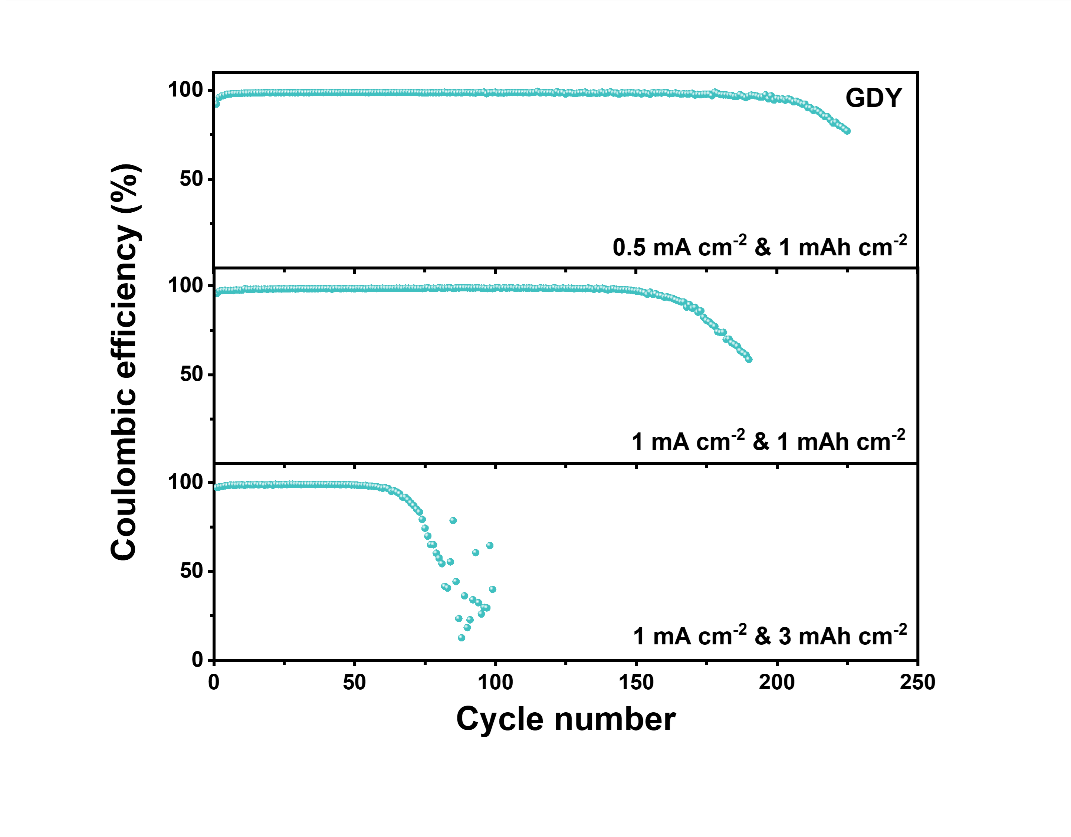


**Fig. S30** Coulombic efficiency of Li-Cu cell equipped with GDY decorated separator at various current densities and areal capacities


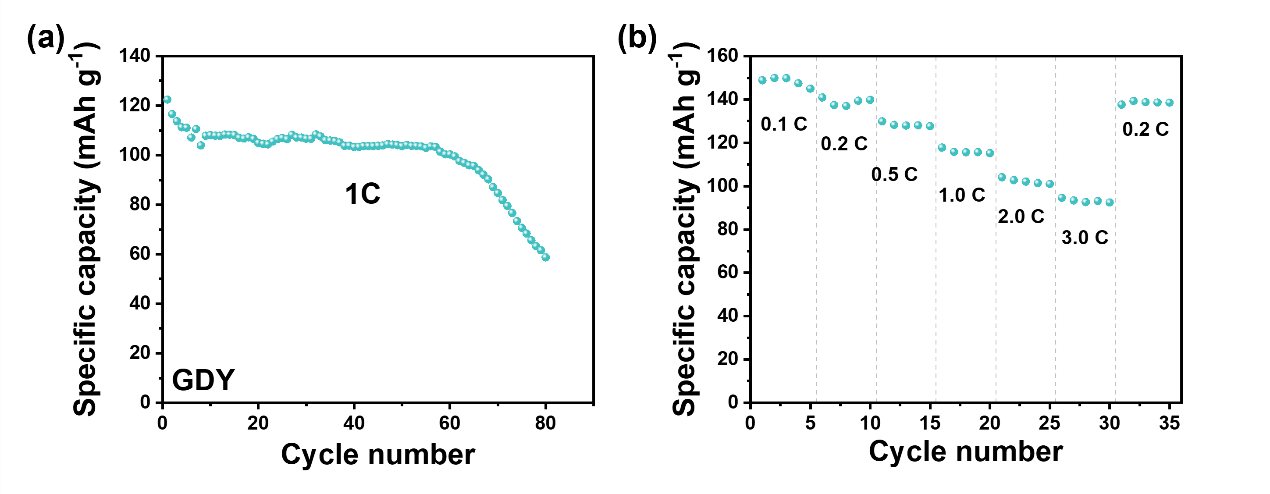


**Fig. S31** Cycle (**a**) and rate (**b**) performances of Li-LFP full cells with GDY decorated separator


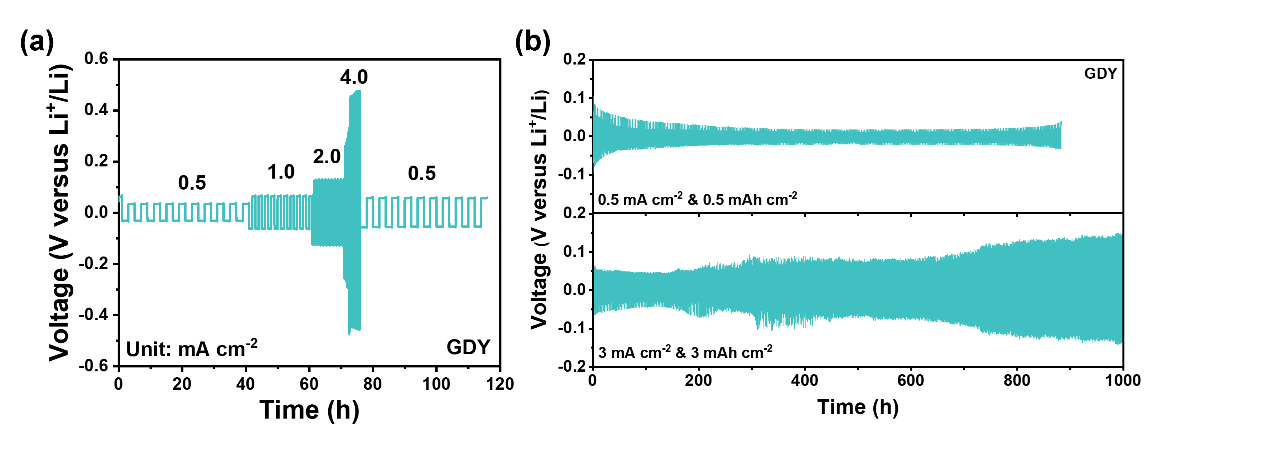


**Fig. S32** Rate (**a**) and cycle (**b**) performances of Li-Li symmetric cells with GDY decorated separator

**Supplementary References**

1. P. Zhai, L. Liu, Y. Wei, J. Zuo, Z. Yang et al. Self-healing nucleation seeds induced long-term dendrite-free lithium metal anode. Nano Lett. **21**, 7715-7723 (2021). <https://doi.org/10.1021/acs.nanolett.1c02521>
2. Q. An, H.-e. Wang, G. Zhao, S. Wang, L. Xu et al. Understanding dual-polar group functionalized COFs for accelerating Li-ion transport and dendrite-free deposition in lithium metal anodes. Energy Environ. Mater. **6**, e12345 (2023). <https://doi.org/10.1002/eem2.12345>
3. Q. Zhao, R. Zhou, C. Wang, J. Kang, Q. Zhang et al. Anion immobilization enabled by cation-selective separators for dendrite-free lithium metal batteries. Adv. Funct. Mater. **32**, 2112711 (2022). <https://doi.org/10.1002/adfm.202112711>
4. G. Lin, K. Jia, Z. Bai, C. Liu, S. Liu et al. Metal-organic framework sandwiching porous super-engineering polymeric membranes as anionphilic separators for dendrite-free lithium metal batteries. Adv. Funct. Mater. **32**, 2207969 (2022). <https://doi.org/10.1002/adfm.202207969>
5. K. H. Han, J. Y. Seok, I. H. Kim, K. Woo, J. H. Kim et al. A 2D ultrathin nanopatterned interlayer to suppress lithium dendrite growth in high-energy lithium-metal anodes. Adv. Mater. **34**, 2203992 (2022). <https://doi.org/10.1002/adma.202203992>
6. W. Wang, A. C. Y. Yuen, Y. Yuan, C. Liao, A. Li et al. Nano architectured halloysite nanotubes enable advanced composite separator for safe lithium metal batteries. Chem. Eng. J. **451**, 138496 (2023). <https://doi.org/10.1016/j.cej.2022.138496>
